# The Interaction between DNMT1 and High‐Mannose CD133 Maintains the Slow‐Cycling State and Tumorigenic Potential of Glioma Stem Cell

**DOI:** 10.1002/advs.202202216

**Published:** 2022-07-07

**Authors:** Yuanyan Wei, Qihang Chen, Sijing Huang, Yingchao Liu, Yinan Li, Yang Xing, Danfang Shi, Wenlong Xu, Weitao Liu, Zhi Ji, Bingrui Wu, Xiaoning Chen, Jianhai Jiang

**Affiliations:** ^1^ NHC Key Laboratory of Glycoconjuates Research Department of Biochemistry and Molecular Biology School of Basic Medical Sciences Fudan University Shanghai 200032 P. R. China; ^2^ Department of Neurosurgery Shandong Provincial Hospital Affiliated to Shandong First Medical University Jinan Shandong 250021 P. R. China; ^3^ Division of Neurosurgery Zhongshan Hospital Fudan University Shanghai 200032 P. R. China

**Keywords:** CD133, DNMT1, glioma stem cell, high mannose, quiescence

## Abstract

The quiescent/slow‐cycling state preserves the self‐renewal capacity of cancer stem cells (CSCs) and leads to the therapy resistance of CSCs. The mechanisms maintaining CSCs quiescence remain largely unknown. Here, it is demonstrated that lower expression of MAN1A1 in glioma stem cell (GSC) resulted in the formation of high‐mannose type *N‐*glycan on CD133. Furthermore, the high‐mannose type *N‐*glycan of CD133 is necessary for its interaction with DNMT1. Activation of p21 and p27 by the CD133–DNMT1 interaction maintains the slow‐cycling state of GSC, and promotes chemotherapy resistance and tumorigenesis of GSCs. Elimination of the CD133–DNMT1 interaction by a cell‐penetrating peptide or MAN1A1 overexpression inhibits the tumorigenesis of GSCs and increases the sensitivity of GSCs to temozolomide. Analysis of glioma samples reveals that the levels of high‐mannose type *N‐*glycan are correlated with glioma recurrence. Collectively, the high mannose CD133–DNMT1 interaction maintains the slow‐cycling state and tumorigenic potential of GSC, providing a potential strategy to eliminate quiescent GSCs.

## Graphical abstract

The lower expression of MAN1A1 results in the formation of high‐mannose type *N‐*glycan of CD133 in GSCs. The interaction between high‐mannose CD133 and DNMT1 blocks the nuclear translocation of DNMT1. Activation of p21 and p27 expression by the CD133–DNMT1 interaction maintains glioma stem cell quiescence, self‐renewal, and tumorigenesis.

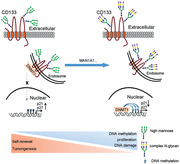

## 1 Introduction

Cancer stem cells (CSCs) are thought to be responsible for tumor growth.^[^ ^1^ ^]^ For example, CD133+ glioma cells form neurospheres, display multilineage differentiation capabilities in vitro, and are highly tumorigenic in the brains of immunocompromised mice.^[^ ^2^ ^]^ Exploring the mechanism of the highly tumorigenic ability and therapy resistance of glioma stem cells (GSCs), contributes to a therapeutic approach for the treatment of brain tumors.

CSCs are maintained in vivo in a quiescent or slow‐growing state. The quiescent state of CSCs protects them from DNA damage.^[^ ^3^, ^4^ ^]^ Conventional chemotherapy and radiation target cells undergoing DNA replication and are therefore not effective against quiescent cells.^[^ ^5^, ^6^, ^7^ ^]^ Therefore, the development of therapeutic approaches that target quiescent CSCs is expected to have a profound impact on cancer eradication.^[^ ^7^ ^]^ For example, abrogation of quiescence in leukemia‐initiating cells by Fbxw7 ablation increased their sensitivity to imatinib.^[^ ^8^ ^]^ Activation of leukemia stem cells by CXCL12 deletion LSC elimination by tyrosine kinase inhibitor treatment.^[^ ^9^ ^]^ Although the mechanisms by which CSCs initiate tumor growth have been gradually explored,^[^ ^10^, ^11^, ^12^ ^]^ the mechanisms of CSCs maintaining quiescence remain largely unknown.

CD133 has been widely used as a marker of CSCs in various tissues.^[^ ^2^, ^13^, ^14^ ^]^ Increasing evidence has shown that CD133 regulates chemotherapy, tumorigenesis, cell differentiation, and migration. For example, CD133 can recruit HDAC6 to deacetylate *β*‐catenin to activate *β*‐catenin signaling.^[^ ^15^ ^]^ Our previous studies have shown that the interaction between CD133 and the PI3K regulatory subunit p85 can activate the PI3K/Akt pathway to promote tumorigenesis of GSCs.^[^ ^16^ ^]^ In addition, activation of FAK by the interaction between CD133 and Src promoted the migration of tumor cells.^[^ ^17^ ^]^ Collectively, CD133 is a functional marker of CSCs. Therefore, clarifying CD133‐ interacting proteins might help to understand the mechanisms of CSCs maintaining quiescence.

Although CD133 has been a potential target for cancer treatment,^[^ ^18^ ^]^ the structural ambiguity of *N‐*glycan of CD133 limits its application in the isolation and elimination of CSCs. CD133 is a highly glycosylated membrane glycoprotein and contained nine *N‐*linked glycosylation sites.^[^ ^19^ ^]^ AC133 antibody, which is used to isolate stem cell, is widely reported to bind the glycosylated epitopes on CD133.^[^ ^20^, ^21^ ^]^ More importantly, during CSC differentiation, the *N‐*glycan structure of CD133, but not CD133 protein or mRNA, was changed.^[^ ^22^ ^]^ Therefore, the glycosylation status of CD133 is closely related to the cell differentiation. However, the structure of *N‐*linked glycan of CD133 in CSCs remains uncovered. Here, we found that the structure of *N‐*glycan of CD133 in GSC is high‐mannose type. The high‐mannose CD133 maintains the slow‐cycling state and tumorigenic potential of GSCs through inhibiting the nuclear translocation of DNA methyltransferase 1 (DNMT1). DNMT1, a member of the DNA methyltransferase family, is responsible for maintaining methylation patterns located in CG dinucleotide‐rich regions.^[^ ^23^ ^]^ DNMT1 regulates chromatin organization, DNA repair, cell cycle regulation, and apoptosis.^[^ ^24^ ^]^ The contributions of DNMT1 to CSCs have been extensively studied. Dnmt1 is essential for the maintenance of leukemia stem cells and mammary and CSC. Conversely, DNMT1 knockdown induces EMT and cancer stem‐like phenotypes in prostate cancer.^[^ ^25^ ^]^ Down‐regulation of DNMT1 increases self‐renewal potential of hepatoma cells.^[^ ^26^ ^]^ Our finding proves that the high mannose CD133–DNMT1 interaction maintains the slow‐cycling state and tumorigenic potential of GSC, providing a potential strategy to eliminate quiescent GSCs.

## 2 Results

### 2.1 CD133 Interacts with DNMT1 In Vitro and In Vivo

**Figure 1:**
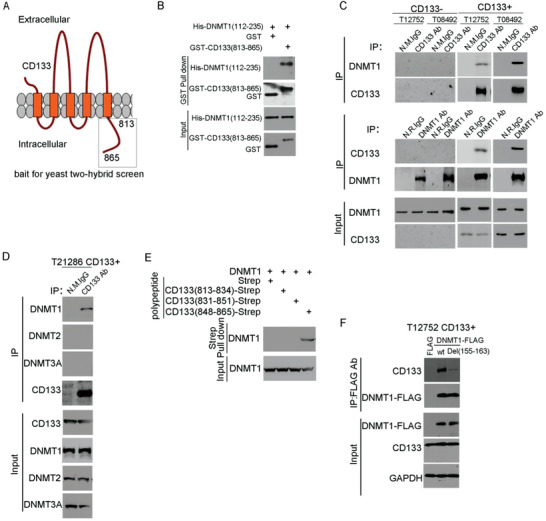
CD133 interacts with DNMT1 depending on its C‐terminal cytoplasmic domain. A) Graphic representation of the proposed structural model of CD133. This protein is modeled as having an extracellular N terminus, a cytoplasmic C terminus, two small cytoplasmic loops, and two large extracellular loops. C‐terminal cytoplasmic domain of CD133 (residues 813–865) (indicated by square frame) is used as the bait for yeast two‐hybrid screen. B) In vitro interaction between CD133 and DNMT1. GST or GST‐CD133(813–865) proteins are incubated with purified His‐DNMT1 (112–235) protein. The GST pull‐down products are blotted with anti‐GST and anti‐His antibodies. C) CD133 interacts with DNMT1 in vivo. The lysates of CD133+ cells and CD133‐ cells isolated from glioblastoma samples are subjected to IP using anti‐CD133 (Clone W6B3C1) or anti‐DNMT1 antibodies, followed by immunoblotting (IB) with anti‐CD133 or anti‐DNMT1 antibodies. Whole‐cell lysates are analyzed by IB with anti‐CD133 or anti‐DNMT1 antibodies as input. D) The interaction between CD133 and the members of DNMT is examined by Co‐IP assay. Lysates of CD133+ cells are subjected to IP using anti‐CD133 antibody (Clone W6B3C1), followed by IB with anti‐CD133 (Clone W6B3C1), anti‐DNMT1, anti‐DNMT2, or anti‐DNMT3A antibodies. Whole‐cell lysates are analyzed by IB with anti‐CD133, anti‐DNMT1, anti‐DNMT2, or anti‐DNMT3A antibodies as input. E) Strep peptide or strep‐tagged CD133 c‐terminal deletion mutant are incubated with purified DNMT1 protein. The Strep pull‐down products are blotted with anti‐DNMT1 antibody. F) The lysates of CD133+ cells expressing FLAG or DNMT1‐FLAG or DNMT1(Del(155–163)) are subjected to IP using anti‐FLAG antibody, followed by IB with anti‐FLAG, or anti‐CD133 antibodies (Clone W6B3C1). Whole‐cell lysates are analyzed by IB with anti‐CD133 (Clone W6B3C1), anti‐FLAG, or anti‐GAPDH antibodies as input.

We used a yeast two‐hybrid screen in an attempt to identify CD133‐ interacting proteins. The C‐terminal cytoplasmic domain of CD133 (residues 813–865) was used as the bait (**Figure** **1**A). The cDNA encoding C‐terminal cytoplasmic domain of CD133 (residues 813–865) was cloned into pGBKT7 vector and was used as the bait to screen pACT2‐human cDNA libraries (human fetal brain). We isolated 6 positive clones from 1 × 10^6^ clones of a human fetal brain library. Among the positive clones, we identified 3 encoding partial sequences of DNMT1 (112–235) (Table S1, Supporting Information). The interaction of CD133 (residues 813–865) with DNMT1 (112–235) in vitro was confirmed by GST pull‐down assay (Figure 1B). To validate the physical interaction between CD133 and DNMT1 in vivo, we isolated CD133+ and CD133‐ cells from human glioblastoma samples (T21286, T12752, and T08492; pathological data see Table S2, Supporting Information) as previously described (Figure S1A, Supporting Information).^[^ ^2^, ^27^ ^]^ CD133+ tumor cells showed characteristics consistent with CSCs: namely, neurosphere formation (Figure S1B, Supporting Information); multilineage differentiation with markers for astrocytes (GFAP), neurons (MAP2) or oligodendrocytes (O4) (Figure S1C, Supporting Information), and expression of stem cell markers Nestin and Sox2 (Figure S1C,D, Supporting Information). CD133+ tumor cells were highly tumorigenic in the brains of immunocompromised mice, and CD133‐ cells did not form detectable tumor even when implanted at 5 × 10^5^ cells per mouse, except for occasional small tumor from a single xenograft source (Figure S1E–G, Supporting Information). Endogenous DNMT1 interacted with endogenous CD133, as shown by reciprocal co‐IP assays (Figure 1C and Figure S1H, Supporting Information). However, CD133 did not interact with other DNMT family members, including DNMT3A or DNMT2 (Figure 1D).

Next, we searched the region responsible for the interaction between CD133 and DNMT1. By strep pull‐down assay, CD133 C‐terminal segment (amino acids 848–865) interacted with DNMT1 in vitro (Figure 1E). A co‐IP assay in CD133+ glioma cells expressing CD133 shRNA and either shRNA‐resistant wild‐type CD133 or its deletion mutant, further showed that the CD133(1–862) mutant lacking a region between residues 863 and 865 could not interact with DNMT1 (Figure S1I, Supporting Information). In a yeast two‐hybrid system, amino acids 112–235 of DNMT1 interacted with the CD133 C‐terminus (Table S1, Supporting Information). Co‐IP assays in CD133+ cells expressing FLAG‐tagged DNMT1 deletion mutants showed that the deletion of a region between DNMT1 residues 155 and 163 reduced the interaction between DNMT1 and CD133 (Figure 1F and Figure S1J, Supporting Information). To examine the interaction between full‐length CD133 and DNMT1 in vitro, strep‐tagged CD133 protein and its mutant Del(836–865) was purified by Strep‐Tactin affinity. By Coomassie blue staining, the purity of CD133 purified protein was over 90% (Figure S1K, Supporting Information). By Strep pull‐down assay, deletion of CD133 amino acids 848–865 reduced the interaction between CD133 and DNMT1 in vitro (Figure S1L, Supporting Information). By enzyme‐linked immunosorbent assay (ELISA), CD133 bound to DNMT1 with Kd = ∼150 nM (Figure S1M, Supporting Information). Together, CD133 interacts with DNMT1 in GSCs depending on its C‐terminal cytoplasmic domain.

### 2.2 The Interaction Between CD133 and DNMT1 Inhibits the Nuclear Translocation of DNMT1

**Figure 2:**
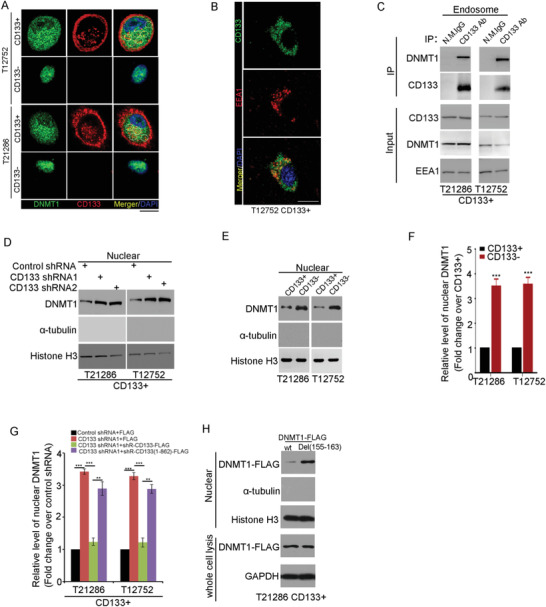
The CD133–DNMT1 interaction inhibits the nuclear translocation of DNMT1. A) Co‐localization of CD133 and DNMT1 is assessed by immunofluorescence staining of CD133 (red) and DNMT1 (green) in T12752 (upper panel) and T21286 (lower panel) CD133+ cells. Nuclei (blue) are counterstained with DAPI. Co‐localization of CD133 and DNMT1 is demonstrated by yellow fluorescence. The interaction between DNMT1 and cytoplasmic CD133 is indicated by dashed circle. Scale bars, 10 µM. B) Co‐localization of CD133 and EEA1 is assessed by immunofluorescence staining in CD133+ cells. Nuclei (blue) are counterstained with DAPI. Co‐localization of CD133 and EEA1 is demonstrated by yellow fluorescence. Scale bars, 10 µM. C) CD133 interacts with DNMT1 at endosome. Lysates of endosome protein isolated from CD133+ glioma cells are subjected to IP using anti‐CD133 antibody (Clone W6B3C1), followed by IB with anti‐CD133 (Clone W6B3C1) or anti‐DNMT1 antibodies. The efficiency of endosome isolation is checked by EEA1 (endosome marker). D) Nuclear distribution of DNMT1 in CD133+ cells expressing control shRNA, CD133 shRNA1, or CD133 shRNA2 is determined by IB. Histone H3 is used as the nuclear marker, and *α*‐tubulin is used as the cytosolic marker. E,F) The level of nuclear DNMT1 in CD133+ cells and matched CD133‐ cells is determined by IB. Histone H3 is used as the nuclear marker, and *α*‐tubulin is used as the cytosolic marker. E. The figures are presented out of three independent experiments. F) The relative densities of DNMT1 to Histone H3 are quantified using densitometry. Values are normalized to that of CD133+ cells. Results are expressed as mean ± SD from three independent experiments; *t* test, ***p < 0.001, Student's *t*‐test. G) The level of nuclear DNMT1 in CD133+ cells expressing control shRNA, CD133 shRNA1, CD133 shRNA1 + shRNA‐resistant wild‐type CD133, or CD133 shRNA1 + shRNA‐resistant CD133(1–862) mutant is determined by IB. Histone H3 is used as the nuclear marker, and *α*‐tubulin is used as the cytosolic marker. The relative densities of DNMT1 to Histone H3 are quantified using densitometry. Values are normalized to that of cells expressing control shRNA. Results are expressed as mean ± SD from three independent experiments; ***p < 0.001, Student's *t*‐test. H) The level of nuclear DNMT1‐FLAG in CD133+ cells expressing FLAG‐DNMT1 or DNMT1(Del(155–163)) is determined by IB. Histone H3 is used as the nuclear marker, and *α*‐tubulin is used as the cytosolic marker.

CD133 is mainly located on the cell surface.^[^ ^28^ ^]^ DNMT1 is usually located in the nucleus.^[^ ^24^ ^]^ We presumed that CD133 might regulate the nuclear localization of DNMT1. By immunofluorescence analysis, exogenous CD133‐GFP co‐localized with DNMT1‐dsRed in the cytoplasm in CD133+ cells (Figure S2A, Supporting Information). Immunofluorescence analysis showed the colocalization between endogenous CD133 and DNMT1 in the cytoplasm in CD133+ cells (**Figure** **2**A), and in GBM tissues (Figure S2B, Supporting Information). Cytoplasmic CD133 is partly located in endosome.^[^ ^29^ ^]^ Consistent with this finding, cytoplasmic CD133 in CD133+ glioma cells colocalized with the endosome marker EEA1 (Figure 2B), not with the Golig marker GM130 (Figure S2C, Supporting Information). By immunoprecipitation from the lysis of endosome from CD133+ cells, DNMT1 associated with CD133 at the endosomes of CD133+ cells (Figure 2C). Thus, DNMT1 interacts with endosomal CD133.

CD133 knockdown did not change the expression level of DNMT1 mRNA or DNMT1 protein (Figure S2D,E, Supporting Information). CD133 knockdown increased the nuclear translocation of DNMT1 in CD133+ glioma cells without obviously changing the nuclear translocation of DNMT3a or DNMT3b (Figure 2D and Figure S2F, Supporting Information). The nuclear translocation of DNMT1 was decreased in CD133+ glioma cells compared to CD133‐ glioma cells (Figure 2E,F). Thus, CD133 inhibits DNMT1 nuclear translocation. To explore the significance of the CD133–DNMT1 interaction in CD133‐ reduced DNMT1 nuclear translocation, CD133+ glioma cells were expressed CD133 shRNA and either shRNA‐resistant wild‐type CD133 or shRNA‐resistant CD133(1–862) mutant. The nuclear translocation of CD133‐binding deficient DNMT1 mutant (Del(155–163) was markedly enhanced, compared to wild type DNMT1 (Figure 2H). Ectopic expression of shRNA‐resistant wild‐type CD133, but not expression of the CD133(1–862) mutant, restored the effect of CD133 knockdown on the nuclear translocation of DNMT1 (Figure 2G and Figure S2I, Supporting Information). Deletion of the DNMT1 region between aa 155 and 163, which is essential for the interaction between CD133 and DNMT1, significantly increased the nuclear localization of DNMT1 in CD133+ cells (Figure 2H). Thus, the CD133–DNMT1 interaction inhibits the nuclear translocation of DNMT1.

### 2.3 The CD133–DNMT1 Interaction Induces the Transcription of Genes Inhibiting the G1/S Transition

**Figure 3:**
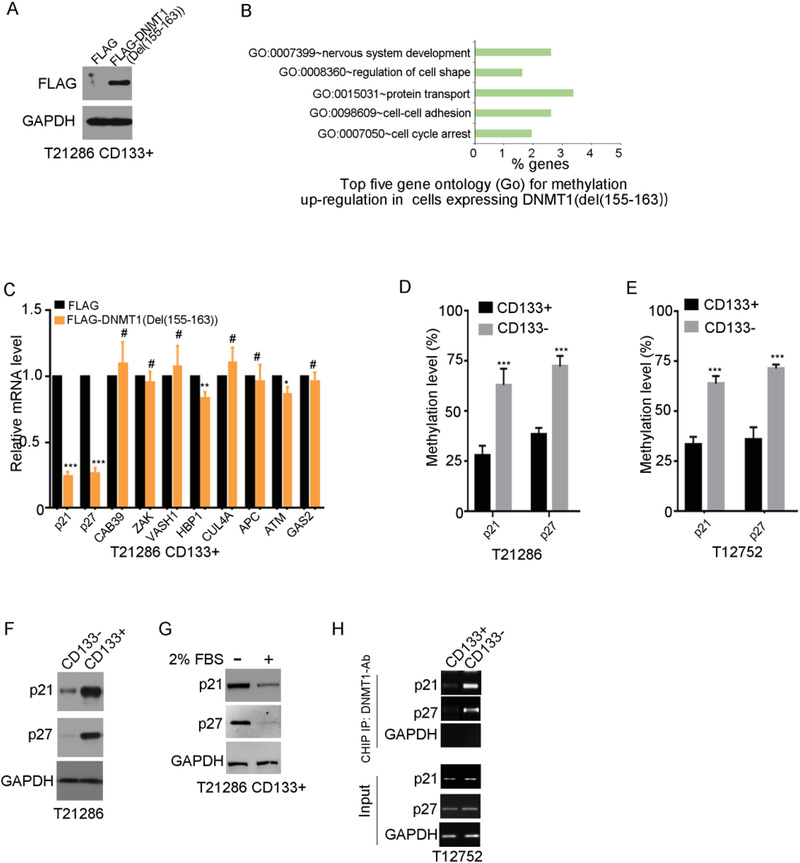
The CD133–DNMT1 interaction upregulates p21 and p27. A) Western blot analysis of FLAG‐DNMT1 in CD133+ cells expressing FLAG or FLAG‐DNMT1(Del(155–163)). GAPDH is used as a loading control. B) By Infinium MethylationEPIC BeadChip arrays, the methylation of 680 annotated genes is increased in CD133+ cells expressing DNMT1 (Del(155–163)) compared to control cells (*p* < 0.001, Δ*β* ≥ 0.15). Gene ontology results (top five, according to *p* value) for 680 genes in which methylation is upregulated in CD133+ cells expressing FLAG‐DNMT1(Del(155–163)) are shown. C) qRT‐PCR quantification of the indicated gene mRNA levels in T21286 CD133+ cells expressing FLAG or FLAG‐DNMT1(Del(155–163)). Data is shown as mean ± SD from three independent experiments; ***p < 0.001, **p < 0.01, *p < 0.05, #*p* > 0.05, Student's t‐test. D‐E) The methylation rate of p21 and p27 promoters in CD133+ cells and matched CD133‐ cells from T21286 (D) and T12752 (E) are analyzed by bisulfite sequencing. Methylation levels are determined by the ratio of converted C nucleotides to total C nucleotides following bisulfite treatment under CpG island. Results are expressed as mean ± SD from three independent experiments; ***p < 0.001, Student's *t*‐test. F) Western blot analysis of p21 and p27 expression in T21286 CD133+ cells and CD133‐ cells. GAPDH is used as a loading control. G) Western blot analysis of p21 and p27 expression in T21286 CD133+ cells treated with 2% FBS for 7 days. GAPDH is used as a loading control. H) Chromatin immunoprecipitation (ChIP) assay is performed in CD133+ cells and CD133‐ cells using a DNMT1 specific antibody, followed by PCR amplification of p21 and p27 promoter regions between +250 to position −100. Chromatin (defined as input) and GAPDH products immunoprecipitated by DNMT1 Ab are used as positive and negative control.

Nuclear DNMT1 catalyzes DNA methylation, thereby inhibiting gene expression.^[^ ^30^ ^]^ We first compared the level of 5‐methylcytosine (5‐mC) between CD133+ cells with matched CD133‐ cells using a 5‐methylcytosine DNA ELISA kit. CD133+ glioma cells displayed lower level of 5‐methylcytosine than matched CD133‐ glioma cells (Figure S3A, Supporting Information). To search for potential DNMT1 target genes in CD133+ cells, CD133‐ binding deficient mutant DNMT1 (Del(155–163)) was transfected into CD133+ cells (**Figure** **3**A). By Infinium MethylationEPIC BeadChip Arrays (850000 CpG sites), 680 annotated genes in CD133+ cells expressing DNMT1 (Del(155–163)) with significant methylation in either their promoter, exons, or introns were identified. GO analysis indicates that the significantly changed genes were involved in the cell cycle arrest, cell adhesion, and nervous system development (Figure 3B and Table S3, Supporting Information). The methylation of genes inhibiting cell cycle progression (including p21/CDKN1A, p27/CDKN1B, HBP1, ATM, APC) are upregulated in CD133+ cells expressing DNMT1 (Del(155–163)) (Table S4, Supporting Information). qRT‐PCR analysis showed that the expression of p21 and p27 was decreased in CD133+ cells expressing DNMT1 (Del(155–163)) (Figure 3C). Overexpression of DNMT1 (Del(155–163)) increased the levels of p21 and p27 promoter methylation (Figure S3B, Supporting Information). In line with these findings, the methylation levels of p21 and p27 promoters were lower in CD133+ cells than in CD133‐ cells by bisulfite sequencing (Figure 3D,E). The expression of p21 and p27 was higher in CD133+ cells than in CD133‐ cells (Figure 3F). Serum reduced the expression of p21 and p27 (Figure 3G). P21 and p27 were mainly expressed in the nuclear of CD133+ cells (Figure S3C, Supporting Information). By Chromatin Immunoprecipitation (ChIP), the binding of DNMT1 to p21 and p27 promoters was increased in CD133‐ cells compared to CD133+ cells (Figure 3H). Accordingly, the methylation levels of p21 and p27 promoters, are lower in GSCs.

Next, a series of experiments were performed to examine the contribution of the CD133–DNMT1 interaction to high level of p21 and p27 in GSCs. First, CD133 knockdown increased the level of 5‐methylcytosine in CD133+ glioma cells (Figure S3D). Ectopic expression of shRNA‐resistant wild‐type CD133, but not that of the CD133(1–862) mutant, restored the effect of CD133 knockdown on the level of 5‐methylcytosine (Figure S3E, Supporting Information). Second, ectopic expression of shRNA‐resistant wild‐type CD133, but not that of the CD133(1–862) mutant, restored the inhibitory effect of CD133 knockdown on the promoter methylation of p21 and p27 (Figure S3F, Supporting Information). Third, ectopic expression of shRNA‐resistant wild‐type CD133, but not that of the CD133(1–862) mutant, eliminated the effects of CD133 knockdown on the expression of p21 and p27 (Figure S3G, Supporting Information). Finally, downregulation of DNMT1 by shRNA (Figure S3H), restored the effect of CD133 knockdown on the promoter methylation of p21 and p27 (Figure S3I, Supporting Information). Together, CD133 upregulates p21 and p27 depending on its interaction with DNMT1.

### 2.4 Nuclear localization of DNMT1 Inhibits the Self‐Renewal Capacity and Tumorigenesis of GSCs

P21 and p27 maintain the quiescence and self‐renewal capacity of stem cells.^[^ ^31^, ^32^, ^33^ ^]^ P21 and p27 promote the association of CDK4 with the D‐type cyclins, inhibit the kinase activity of CDK4 i which could phosphorylate Rb protein, and subsequently prevent cell cycle progression.^[^ ^34^ ^]^ Downregulation of p21 or p27 in CD133+ cells (Figure S4A,B, Supporting Information), increased the activity of CDK4 (Figure S4C,D, Supporting Information). Consistent with this finding, the kinase activity of CDK4 was lower in CD133+ cells than in CD133‐ cells (Figure S4E, Supporting Information). By FCS analysis, downregulation of p21 or p27 in CD133+ cells promoted G1/S transition (Figure S4F,G, Supporting Information), and increased the ratio of EdU‐positive cells (Figure S4H, Supporting Information). Quiescence of stem cells acts to limit the accumulation of DNA damage in normal and CSCs.^[^ ^35^ ^]^ *γ*‐H2AX foci are widely used as a marker of DNA damage.^[^ ^36^ ^]^ Downregulation of p21 or p27 increased the ratio of *γ*‐H2AX‐positive cells (Figure S4I, Supporting Information). HSCs remain in quiescence to sustain their long‐term self‐renewal potential.^[^ ^37^ ^]^ The single‐cell neurosphere formation assay is the conventional method to measure the self‐renewal capacity of GSCs.^[^ ^11^, ^27^ ^]^ Down‐regulation of p21 or p27 increased the diameter of spheres at passage 1 (Figure S4J, Supporting Information). However, the number of spheres was significantly decreased at passage 2 (Figure S4J,K, Supporting Information). By the in vivo limiting dilution assay, downregulation of p21 or p27 inhibited the tumorigenesis of CD133+ cells (Figure S4L, Supporting Information). Thus, down‐regulation of p21 or p27 promotes the proliferation and reduces the self‐renewal potential of GSCs.

**Figure 4:**
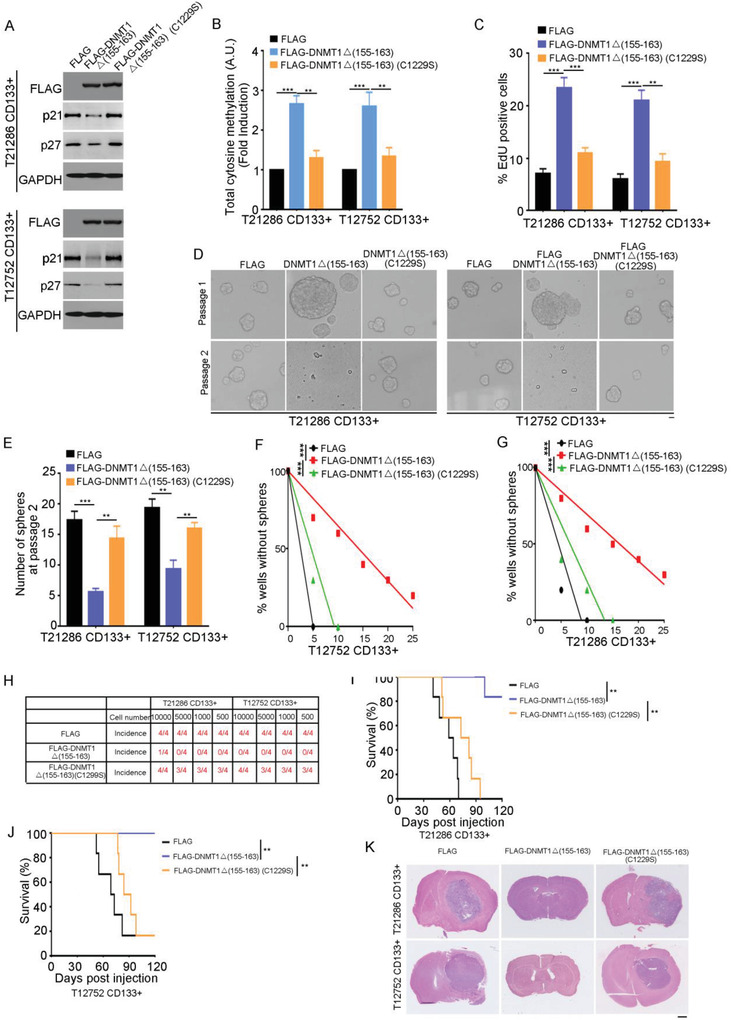
Nuclear localization of DNMT1 inhibits the self‐renewal ability and the tumorigenesis of GSCs. A) Western blot analysis of FLAG‐DNMT1, p21, p27 expression in CD133+ cells expressing FLAG, FLAG‐DNMT1(Del(155–163)), or its C1229S mutant. GAPDH is used as a loading control. B) The level of total 5‐methylcytosine in CD133+ cells expressing FLAG, FLAG‐DNMT1(Del(155–163)) or its C1229S mutant is examined by ELISA kit. Values are normalized to that of cells expressing FLAG. Results are expressed as mean ± SD from three independent experiments; ***p < 0.001, **p < 0.01, Student's *t*‐test. C) Analysis of 5‐ethynyl‐2′‐deoxyuridine (EdU)‐labeled CD133+ cells expressing FLAG, FLAG‐DNMT1(Del(155–163)) or its C1229S mutant. The percentage of EdU‐positive cells is measured. Results are expressed as mean ± SD from six independent experiments; ***p < 0.001, **p < 0.01, Student's *t*‐test. D,E) The number of spheres derived from 100 CD133+ cells expressing FLAG, FLAG‐DNMT1(Del(155–163)), or its C1229S mutant at passages 1 and 2. D) Representative images are shown. E) Results are expressed as mean ± SD from three independent experiments; ***p < 0.001, **p < 0.01, Student's *t*‐test. Scale bar represents 10 µM. F,G) Limiting dilution assay shows overexpression of DNMT1(Del(155–163)) reduced stem cell frequency in T12752 (F) or T21286 (G) CD133+ cells. *n* = 10, ***p < 0.001 by ELDA analysis. H) The tumor‐initiating capacity of CD133+ cells expressing FLAG, FLAG‐DNMT1(Del(155–163)) or its C1229S mutant. An intracranial limiting dilution tumor formation assay (employing 10000, 5000, 1000, and 500 cells per mouse) is performed using CD133+ cells infected with the indicated lentivirus. The table displays the number of mice developing tumors. I–K) T21286 (I) or T12752 (J) CD133+ cells expressing FLAG, FLAG‐DNMT1(Del(155–163)) or its C1229S mutant are implanted into immunocompromised mice brain (5000 cells per mouse). Mice are sacrificed when they are moribund or 120 days after implantation. I,J) Survival of mice (*n* = 6) is evaluated by Kaplan‐Meier analysis (**p < 0.01, log rank test). K) H&E staining of mouse brain shows tumors formation by CD133+ cells expressing FLAG, FLAG‐DNMT1(Del(155–163)) or its C1229S mutant. Scale bar, 1 cm. C1229 (Cys at position 1229 ) in the FLAG‐DNMT1 Del(155‐163) protein corresponds to Cys at position 1226 in wild type human DNMT1 protein.

The catalytic cysteine C1226 at DNMT1 (human) is necessary for its DNA methyltransferase activity.^[^ ^38^, ^39^ ^]^Due to the FLAG tag and the deletion of amino acids, catalytic cysteine at position 1226 was shifted to position 1229 in the FLAG‐DNMT1 Del(155‐163) protein. To evaluate the effect of CD133‐inhibited DNMT1 nuclear localization in GSCs, CD133+ cells were constructed to express FLAG‐tagged DNMT1 Del(155–163) or its C1229S mutant (**Figure** **4**A). Ectopic expression of DNMT1 Del(155–163), but not its C1229S mutant, significantly increased the level of 5‐methylcytosine in CD133+ cells (Figure 4B) and the ratio of EdU‐positive cells (Figure 4C). And, ectopic expression of DNMT1 Del(155–163), but not its C1229S mutant reduced the sphere formation of CD133+ cells at passage 2 (Figure 4D,E), inhibited the stem cell activity by limited dilution assay (Figure 4F,G), and inhibited the tumor‐initiating capacity of CD133+ cells (Figure 4H). Importantly, overexpression of DNMT1 Del(155–163), but not its C1229S mutant, increased the survival of tumor‐bearing mice (Figure 4I–K). We further evaluated whether DNMT1 regulates the quiescence state of CD133+ cells through inhibition of p21 and p27 expression. Ectopic expression of DNMT1 Del(155–163), but not its C1229S mutant increased the G1/S transition in CD133+ cells (Figure S4M,N, Supporting Information). The effect of DNMT1 Del(155–163) on the sphere formation and cell proliferation of CD133+ cells could be partially rescued by ectopic expression of p21 or p27 (Figure S4O,P, Supporting Information). Together, the nuclear localization of DNMT1 inhibits GSC self‐renewal and tumorigenesis, mainly depending on its DNA methylation activity.

### 2.5 CD133 Maintains the Self‐Renewal and Tumorigenesis of GSCs Through Its Interaction with DNMT1

**Figure 5:**
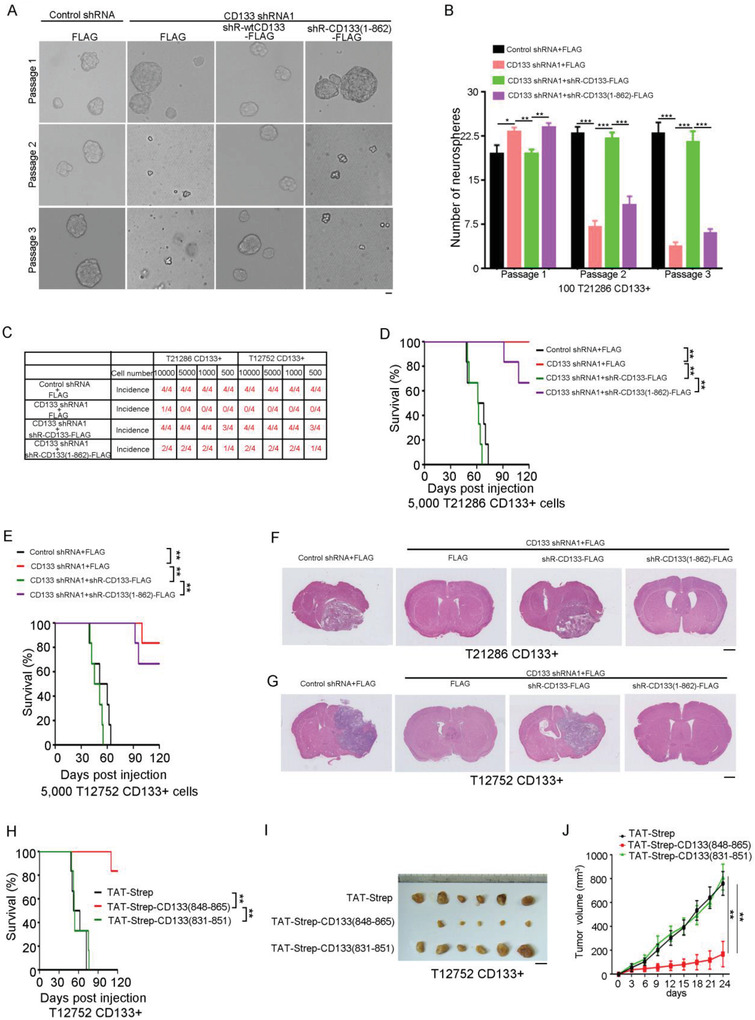
The CD133–DNMT1 interaction maintains the self‐renewal capacity and tumorigenesis of GSC. A,B) Single cell neurosphere formation assay of CD133+ cells expressing control shRNA, CD133 shRNA1, CD133 shRNA1+shRNA‐resistant wild type CD133, or CD133 shRNA1+shRNA‐resistant CD133(1–862) at passages 1–3. A) Representative images of neurosphere are shown. B) The number of neurospheres is shown. Results are expressed as mean ± SD from three independent experiments; ***p < 0.001, **p < 0.01, Student's *t*‐test. Scale bar represents 10 µM. C) Wild type CD133, but not CD133(1–862) mutant, rescues the effect of CD133 knockdown on the tumor‐initiating capacity of CD133+ cells. An intracranial limiting dilution tumor formation assay (employing 10 000, 5000, 1000, and 500 cells per mouse) is performed using CD133+ cells infected with the indicated lentivirus. The table displays the number of mice developing tumors. D–G) CD133+ cells from glioblastoma specimen T21286 (D,F) or T12752 (E,G) expressing Control shRNA, CD133 shRNA1, CD133 shRNA1+shRNA‐resistant wild type CD133, or CD133 shRNA1+shRNA‐resistant CD133(1–862) are implanted into immunocompromised mice brain (5000 cells per mouse). Mice are sacrificed when they are moribund or 120 days after implantation. D,E) Survival of mice (*n* = 6) is evaluated by Kaplan‐Meier analysis (**p < 0.01, log rank test). F,G). H&E staining of mouse brain shows tumors formation by CD133+ cells expressing Control shRNA, CD133 shRNA1, CD133 shRNA1+shRNA‐resistant wild type CD133, or CD133 shRNA1+shRNA‐resistant CD133(1–862). Scale bar, 1 cm. shR, shRNA‐resistant. Scale bar, 1 cM. H) T12752 CD133+ cells treated with the indicated peptides are i implanted into immunocompromised mice brain (5000 cells per mouse). Mice are sacrificed when they are moribund or 120 days after implantation. Survival of mice (*n* = 6) is evaluated by Kaplan‐Meier analysis (**p < 0.01, log rank test). I,J) T12752 CD133+ cells treated with the indicated peptides cells are subcutaneously injected into immunodeficient mice. I) The images of the xenograft of GSC treated with control or peptides. Scale bar, 1 cm. J) Tumor volumes are measured after tumor cell inoculation every three days. Results are expressed as mean ± SD (*n* = 6 mice; **p < 0.01). Student's *t*‐test.

CD133 knockdown impaired the self‐renewal of GSCs.^[^ ^16^, ^40^ ^]^ Supporting this notion, CD133 knockdown inhibited the expression of p21 and p27 (Figure S5A, Supporting Information) and increased pRb phosphorylation (Figure S5B, Supporting Information). And, CD133 knockdown reduced the sphere formation of CD133+ cells (Figure S5C,D, Supporting Information), inhibited the stem cell activity by limited dilution assay (Figure S5E,F, Supporting Information), and reduced the tumor‐initiating capacity of CD133+ cells (Figure S5G, Supporting Information). Next, the self‐renewal and tumorigenic abilities of CD133+ glioma cells expressing CD133 shRNA and either shRNA‐resistant wild‐type CD133 or shRNA‐resistant CD133(1–862) mutant were evaluated. The number of spheres was significantly decreased in passage 2–3 with knockdown of CD133. The inhibitory effect of CD133 knockdown on neurosphere formation was fully rescued by the expression of shRNA‐resistant wild‐type CD133, but not by the shRNA‐resistant CD133(1–862) mutant (**Figure** **5**A,B and Figure S5H, Supporting Information). And, by limited dilution assay, the inhibitory effect of CD133 knockdown on stem cell activity of GSC was fully rescued by the expression of shRNA‐resistant wild‐type CD133, but not by the shRNA‐resistant CD133(1–862) mutant (Figure S5I,J, Supporting Information). Furthermore, ectopic expression of wild‐type CD133, but not the CD133(1–862) mutant, fully rescued inhibitory effect of CD133 depletion on the tumor‐initiating capacity of GSCs (Figure 5C). Importantly, CD133 knockdown increased the survival of tumor‐bearing mice, which was fully restored by expression of shRNA‐resistant wild‐type CD133, but not by the shRNA‐resistant CD133(1–862) mutant (Figure 5D–G). By histochemical staining in xenografts, a reduction in Nestin, CD133, and cytoplasmic DNMT1 in xenografts formed by CD133+ cells expressing CD133 shRNA could be rescued by ectopic expression of wild‐type CD133, not by expression of the CD133(1–862) mutant (Figure S5K, Supporting Information). Thus, the CD133–DNMT1 interaction is critical for CD133 sustaining GSC self‐renewal and tumorigenesis.

The significance of the CD133–DNMT1 interaction in GSC maintenance motivated us to identify the inhibitors of CD133–DNMT1 interaction. Cell‐penetrating peptide‐conjugated peptides or proteins can cross cellular membranes and inhibit intracellular protein‐protein interactions.^[^ ^41^, ^42^ ^]^ The sequence from the C‐terminal domain of CD133 interacting with DNMT1 (from amino acids 848 to 865) was fused to the TAT penetrating sequence (YGRKKRRQRRR) (Figure S5L, Supporting Information). The TAT‐strep‐CD133(848–865) peptide translocated into GSCs (Figure S5M, Supporting Information), and inhibited the interaction between CD133 and DNMT1 (Figure S5N, Supporting Information). Furthermore, the TAT‐strep‐CD133(848–865) peptide reduced the neurosphere formation in GSCs (Figure S5O, Supporting Information). More importantly, the TAT‐strep‐CD133(848–865) peptide reduced the tumor‐initiating capacity of GSCs (Figure S5P, Supporting Information), and increased the survival of tumor‐bearing mice (Figure 5H and Figure S5Q, Supporting Information). And, the TAT‐strep‐CD133(848–865) peptide reduced the growth in vivo of GSCs (Figure 5I,J). Together, inhibition of the CD133–DNMT1 interaction reduces GSC self‐renewal and tumorigenesis.

### 2.6 The CD133–DNMT1 Interaction Maintains GSC Slow‐Cycling State

**Figure 6:**
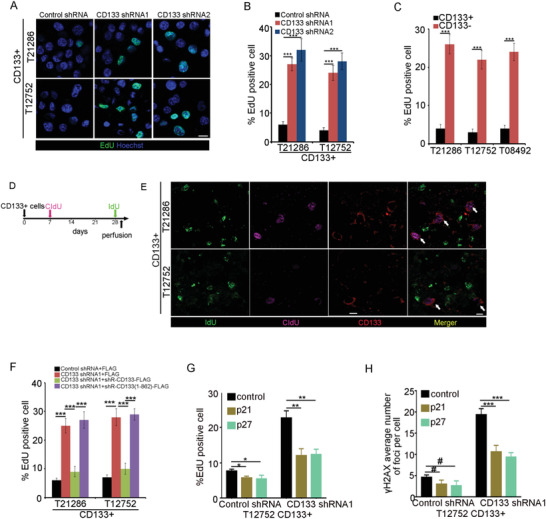
The CD133–DNMT1 interaction maintains the quiescence of GSC. A,B) Immunofluorescence analysis of EdU‐labeled CD133+ cells expressing control shRNA, CD133 shRNA1, or CD133 shRNA2. A) Representative images of immunofluorescence are shown. B) The percentage of EdU‐positive cells is measured. Results are expressed as mean ± SD from six independent experiments; ***p < 0.001, Student's *t*‐test. Scale bar represents 10 µM. C) Analysis of the percentage of EdU‐positive cells in CD133+ cells and CD133‐ cells. Results are expressed as mean ± SD from six independent experiments; ***p < 0.001, Student's *t*‐test. D,E) In vivo CIdU and IdU incorporation assay is used to examine the proliferation of GSCs. D. CD133+ cells are intracranially implanted into immunocompromised mice brain. 7 days later, mice are intraperitoneally injected with CIdU. After 4 weeks, mice are intraperitoneally injected with IdU. 24 h later, mice are sacrificed and perfused. E) Glioblastoma orthotropic xenograft is assessed by immunofluorescence staining of CD133 (red), IdU (green), and CIdU (purple). White arrow indicates the CD133+ cells. Scale bar represents 10 µM. F) Immunofluorescence analysis the percentage of EdU‐positive cells in CD133+ cells expressing control shRNA, CD133 shRNA1, CD133 shRNA1+shR CD133, or CD133 shRNA1+shR CD133(1–862). Results are expressed as mean ± SD from six independent experiments; ***p < 0.001, Student's *t*‐test. shR, shRNA‐resistant. G) Analysis of the percentage of EdU‐positive cells in CD133+ cells expressing control shRNA or CD133 shRNA1 and control or p21 or p27. Results are expressed as mean ± SD from six independent experiments; **p < 0.01, *p < 0.05, Student's *t*‐test. H) Immunofluorescence analysis of *γ*H2AX foci formation in CD133+ cells expressing control shRNA or CD133 shRNA1 and control or p21 or p27. The number of *γ*H2AX foci‐positive cells is measured. Results are expressed as mean ± SD from three independent experiments; #*p* > 0.05, ***p < 0.001, Student's t‐test.

The contribution of the CD133–DNMT1 interaction to GSC quiescence was next investigated. By EdU incorporation assay, CD133 knockdown exhibited a 3‐ to 4‐fold increase in EdU incorporation compared to the control cells (**Figure** **6**A,B). CD133‐ glioma cells exhibited a 4‐ to 5‐fold increase in EdU incorporation compared to CD133+ glioma cells (Figure 6C). FACS analysis further showed that downregulation of CD133 promoted the G1/S transition (Figure S6A, Supporting Information). We next performed CIdU and IdU incorporation assays to confirm the quiescence state of GSCs in vivo. CD133+ glioma cells were transplanted into mouse brain and CldU and IdU were injected 7 days and 28 days, respectively. The mice were killed 24 h after injection of IdU (Figure 6D). CD133+ glioma cells possessed higher CIdU incorporation and lower IdU incorporation than CD133‐glioma cell (Figure 6E). CD133 knockdown increased *γ*H2AX foci formation in GSCs (Figure S6B,C, Supporting Information). Thus, CD133 downregulation enhances the proliferation and DNA damage of GSCs.

We next performed a series of experiments to examine the contribution of the CD133–DNMT1 interaction to GSC quiescence. First, forced expression of shRNA‐resistant wild‐type CD133, but not of CD133(1–862), rescued the effect of CD133 knockdown on EdU incorporation and G1/S transition (Figure 6F and Figure S6D, Supporting Information). Second, ectopic expression of shRNA‐resistant wild‐type CD133, but not of CD133(1–862) mutant, rescued the positive effect of CD133 knockdown on *γ*H2AX foci formation (Figure S6E, Supporting Information). Third, forced expression of p21 or p27 rescued the effect of CD133 knockdown on the EdU incorporation and *γ*H2AX foci formation of CD133+ cells (Figure 6G,H). Thus, upregulation of p21 and p27 by the CD133–DNMT1 interaction maintains GSC slow‐cycling state.

### 2.7 The CD133–DNMT1 Interaction Promotes the Resistance of GSCs to the Chemotherapeutic Agent Temozolomide

**Figure 7:**
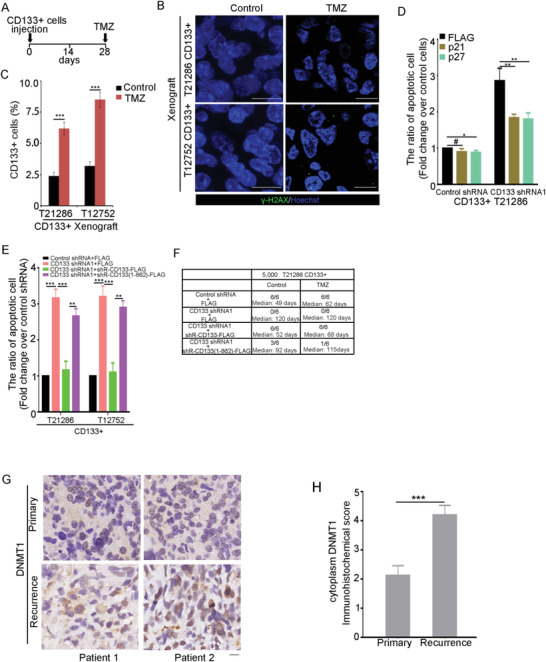
The effect of the CD133–DNMT1 interaction on the apoptosis of GSCs induced by TMZ. A–C) CD133+ cells are intracranially implanted into immunocompromised mice brain. 4 weeks later, temozolomide (TMZ) (2.5 mg kg^−1^) is administered orally every day for 5 days (A). B) Treatment with temozolomide increased the DNA damage in glioma cells. Tissue sections are probed with anti‐*γ*‐H2AX Ab (green), and nuclei are counterstained with Hoechst 33258 (blue). Scale bars, 10 µM. C) The percentage of CD133+ cells in glioblastoma orthotropic xenograft treated with control or temozolomide are measured by flow cytometry. Results are expressed as mean ± SD from three independent experiments; ***p < 0.001, Student's *t*‐test. D) CD133+ cells expressing control shRNA or CD133 shRNA1 and p21 or p27 are treated for 48 h with temozolomide (200 µM). The ratio of apoptotic cells is measured by flow cytometry. Values are normalized to that of cells expressing control shRNA. Results are expressed as mean ± SD from three independent experiments; **p < 0.01, *p < 0.05, #*p* > 0.05, Student's *t*‐test. E) CD133+ cells expressing Control shRNA, CD133 shRNA1, CD133 shRNA1+shRNA‐resistant wild type CD133, or CD133 shRNA1+shRNA‐resistant CD133(1–862) are treated for 48 h with temozolomide (TMZ). The ratio of apoptotic cells is measured by FACS. Values are normalized to that of cells expressing Control shRNA+FLAG. Results are expressed as mean ± SD from three independent experiments; ***p < 0.001, **p < 0.01. Student's *t*‐test. F) The tumor‐initiating capacity of 5000 CD133+ cells expressing control shRNA,CD133 shRNA1, CD133 shRNA1+shRNA‐resistant wild type CD133, or CD133 shRNA1+shRNA‐resistant CD133(1–862) treated with or without TMZ. Mice are sacrificed when they are moribund or 120 days after implantation. The table displays the number of mice developing tumors and the median survival time of mice. G,H) IHC analysis of DNMT1 in 16 paired primary and recurrent glioma sections. G) Representative microphotographs of IHC staining of DNMT1 in primary and recurrent glioma sections. Scale bar represents 10 µM. H) The scores for quantitative staining of cytoplasmic DNMT1 in the tissue sections are determined according to a total score (range, 0–8). Values are mean ± SD (*n* = 16). ***p < 0.001. Student's *t*‐test.

Quiescent CSCs are resistant to conventional chemotherapy and radiation.^[^ ^4^, ^43^, ^44^ ^]^ GSCs are resistant to conventional chemotherapy and radiation.^[^ ^45^, ^46^ ^]^ Next, the contribution of the CD133–DNMT1 interaction to GSC resistance to temozolomide was evaluated. CD133+ glioma cells were transplanted into mouse brain. After 4 weeks, tumor‐bearing mice were orally administered temozolomide (**Figure** **7**A). After temozolomide chemotherapy, the expression of the DNA damage marker *γ*‐H2AX and the apoptosis marker cleaved‐caspase‐3 was obviously increased, indicating the effectiveness of temozolomide treatment (Figure 7B and Figure S7A, Supporting Information). By immunohistochemical (IHC) staining, the level of cytoplasmic DNMT1 was obviously increased after temozolomide treatment (Figure S7B, Supporting Information).

By FACS analysis of CD133 expression in xenografts, temozolomide treatment increased the ratio of CD133+ cells (Figure 7C), indicating that CD133+ cells are resistant to temozolomide. Supporting this point, the ratio of apoptotic cells induced by temozolomide was significantly reduced in CD133+ cells compared to CD133‐ cells (Figure S7C, Supporting Information).

Next, the contribution of the CD133–DNMT1 interaction to GSC resistance to temozolomide was examined. First, CD133 knockdown increased the percentage of apoptotic cells induced by temozolomide (Figure S7D, Supporting Information). Ectopic expression of p21 or p27 rescued the effect of CD133 depletion on the resistance of CD133+ cells to temozolomide (Figure 7D). Second, the inhibitory effect of CD133 knockdown on the resistance of CD133+ cells to temozolomide was fully rescued by the expression of shRNA‐resistant wild‐type CD133, but not by the shRNA‐resistant CD133(1–862) mutant (Figure 7E). Third, TMZ reduced the tumor‐initiating capacity of shRNACD133‐1/CD133+ cell expressing shRNA‐resistant CD133(1–862) mutant, but not shRNA‐resistant wild‐type CD133 (Figure 7F). The TAT‐strep‐CD133(848–865) peptide increased the ratio of apoptotic CD133+ cell induced by temozolomide (Figure S7E, Supporting Information). Resistance to chemotherapeutic agents promotes cancer recurrence.^[^ ^47^, ^48^ ^]^ By IHC staining on paraffin‐embedded sections from paired primary and recurrent tissues, the level of cytoplasmic DNMT1 in recurrent tissues was significantly higher than in primary tissues (Figure 7G,H). Together, the CD133–DNMT1 interaction promotes GSC resistance to the chemotherapeutic agent temozolomide.

### 2.8 The High‐Mannose *N‐*Glycan of CD133 Promotes Its Interaction with DNMT1

**Figure 8:**
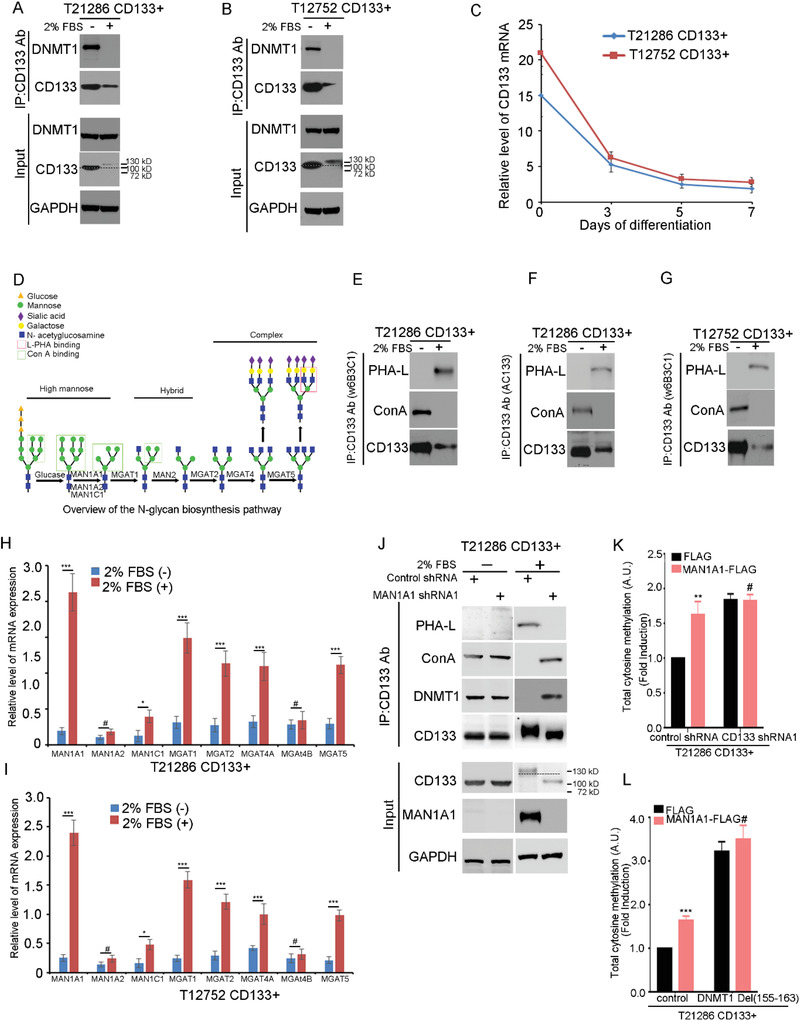
The high‐mannose *N‐*glycan of CD133 promotes the interaction between CD133 and DNMT1. A,B) The interaction between CD133 and DNMT1 during GSC differentiation is examined by Co‐IP assay. The lysates of T21286 (A) and T12752 (B) CD133+ cells treated with 2% FBS for 7 days are subjected to IP using anti‐CD133 (Clone W6B3C1), followed by IB with anti‐CD133 (Clone W6B3C1) or anti‐DNMT1 antibodies. Whole cell lysates are analyzed by IB with anti‐CD133 (Clone W6B3C1) or anti‐DNMT1 antibodies as input. Dotted line indicates the shift of CD133 molecular weight. A molecular‐weight size marker is shown. C) qRT‐PCR quantification of the mRNA levels of CD133 in CD133+ cells from glioblastoma specimens (T21286 and T12752) after treatment with 2% FBS at different time points. Values are mean ± SD from three independent experiments. D) Overview of the *N‐*glycan biosynthesis pathway. Relationships between *N‐*glycans, GlcNAc‐transferases, and plant lectins. The boxed shaded structures are recognized by the plant lectins ConA and PHA‐L. ConA lectin, recognizing high mannose glycans; PHA‐L lectin, recognizing *β*1,6 branched GlcNAc. E–G) Lectin blot is performed to analyze the structure of CD133 *N‐*glycan during GSC differentiation. The lysates of T21286 (E,F) and T12752 (G) CD133+ cells treated with 2% FBS for 7 days are subjected to IP using anti‐CD133 (Clone W6B3C1) Ab (E,G) or anti‐CD133 Ab (Clone AC133) Ab (F), followed by IB with anti‐CD133 antibody or biotinylated lectins. H,I) qRT‐PCR analysis of the mRNA levels of MAN1A1, MAN1A2, MAN1C1, MGAT1, MGAT2, MGAT4A, MGAT4B, or MGAT5 in T21286 (H) and T12752 (I) CD133+ cells treated with or without 2% FBS for 7 days. Values are mean ± SD from three independent experiments; ***p < 0.001, **p < 0.01, #, ns. Student's *t*‐test. J) MAN1A1 regulated the *N‐*glycosylation of CD133. The lysates of CD133+ cells treated with 2% FBS for 7 days transfected with control shRNA or MAN1A1 shRNA are subjected to IP using anti‐CD133 Ab, followed by IB with anti‐CD133 Ab (Clone W6B3C1), anti‐DNMT1 Ab or biotinylated lectin. Whole cell lysates are analyzed by IB with anti‐CD133 (Clone W6B3C1) Ab, anti‐DNMT1 Ab, or anti‐MAN1A1 Ab as input. GAPDH is used as a loading control. Dotted line indicates the shift of CD133 molecular weight. A molecular‐weight size marker is shown. K) The level of total 5‐methylcytosine in CD133+ cells expressing control shRNA, or CD133 shRNA1 and FLAG or MAN1A1‐FLAG is examined by ELISA kit. Values are normalized to that of CD133+ cells. Results are expressed as mean ± SD from three independent experiments; **p < 0.01, #, ns. Student's *t*‐test. L) The level of total 5‐methylcytosine in CD133+ cells expressing FLAG, or MAN1A1‐FLAG and control or DNMT1(Del(155–163)) is examined by ELISA kit. Values are normalized to that of CD133+ cells. Results are expressed as mean ± SD from three independent experiments; ***p < 0.001, #, ns. Student's *t*‐test.

After the differentiation of GSCs, the levels of CD133 protein and mRNA expression were decreased (**Figure** **8**A–C). Interestingly, the molecular weight of CD133 was obviously increased after GSCs differentiation (Figure 8A,B, indicated by dotted line). The change of protein molecular weight frequently results from posttranslational modification. CD133 is a heavily *N‐*glycosylated protein.^[^ ^49^ ^]^ The glycan structure of glycoproteins can be recognized by plant lectins (Figure 8D).^[^ ^50^ ^]^ CD133 immunoprecipitated from GSCs could be recognized by ConA lectin (recognizing high mannose), but not by PHA‐L lectin (recognizing *β*‐1,6 branched *N‐*acetylglucosamine). However, CD133 immunoprecipitated from differentiated cells could be recognized by PHA‐L lectins, but not by Con A lectin (Figure 8E–G). Thus, during the differentiation of GSC, the structure of CD133 *N‐*glycan is converted from the high‐mannose type to the complex type.

MAN1A and *N‐*acetylglucosaminyltransferases (Mgat1, Mgat2, Mgat4A/B and Mgat5) form a linear pathway that initiates the *N‐*acetylglucosamine (GlcNAc) branches on newly synthesized glycoproteins (Figure 8D).^[^ ^51^ ^]^ The mRNA and protein expression levels of MAN1A1, MGAT1, MGAT2, MGAT4A, and MGAT5 were significantly increased after the differentiation of GSCs (Figure 8H,I and Figure S8A, Supporting Information). MAN1A1, cleavages *α*‐1,2‐bound mannose sugars from high‐mannose glycans (Man8‐9GlcNAc2), resulting in 5‐mannose glycans (Man5GlcNAc2) and promotes the conversion of high‐mannose to hybrid and complex *N‐*glycans.^[^ ^52^ ^]^ Knockdown of MAN1A1 by short hairpin RNA decreased the complex *N‐*glycan form of CD133 and increased the high‐mannose form of CD133 (Figure 8J). Thus, lower expression of MAN1A1 is responsible for the formation of high‐mannose type *N‐*glycan of CD133 in GSCs.

*N‐*Glycosylation regulates protein stability and protein delivery to the cell membrane.^[^ ^53^, ^54^ ^]^ Knockdown of MAN1A1 in differentiated cells (Figure S8B, Supporting Information), did not change the delivery of CD133 to the cell surface (Figure S8C, Supporting Information). Knockdown of MAN1A1 in differentiated cells increased the interaction between CD133 and DNMT1 (Figure 8J). Ectopic expression of MAN1A1 in CD133+ cells decreased the interaction between CD133 and DNMT1 (Figure S8D, Supporting Information). Furthermore, MAN1A1 overexpression increased the nuclear location of DNMT1, which could be blocked by CD133 knockdown (Figure S8E, Supporting Information). MAN1A1 overexpression induced the level of 5‐methylcytosine, which could be blocked by CD133 knockdown or CD133‐binding deficient DNMT1 mutant (Figure 8K,L). Collectively, high‐mannose *N‐*glycan inhibits DNA 5‐methylation by maintaining the CD133–DNMT1 interaction.

Next, the contribution of lower MAN1A1 expression to the characteristics of GSCs was evaluated. Ectopic expression of MAN1A1 increased TMZ‐induced CD133+ cells apoptosis, which was obviously blocked by CD133 knockdown (Figure S8F, Supporting Information). MAN1A1 overexpression inhibited the self‐renewal and tumorigenesis of CD133+ cells, which was obviously blocked by CD133 knockdown (Figure S8G,H, Supporting Information). Knockdown of MAN1A1 inhibited serum‐induced differentiation of CD133+ cells (Figure S8I–K, Supporting Information). We isolated cells with high mannose *N‐*glycan (HM) from human glioblastoma samples (T21278, T22456) through magnetic cell sorting using Con A lectin (Figure S8L, Supporting Information). HM+ tumor cells showed characteristics consistent with CSCs, neurosphere formation (Figure S8M, Supporting Information), and expression of the stem cell markers Sox2 (Figure S8N, Supporting Information). HM+ tumor cells were highly tumorigenic in the brains of immunocompromised mice, and HM‐ cells did not form detectable tumor even when implanted at 10^5^ cells (Figure S8O,P, Supporting Information). The ratio of apoptotic cells induced by temozolomide was significantly reduced in HM+ cells compared to HM‐ cells (Figure S8Q, Supporting Information). By IHC staining on paraffin‐embedded sections from paired primary and recurrent tissues, the level of high mannose *N‐*glycan in recurrent tissues was significantly higher than in primary tissues (Figure S8R,S, Supporting Information). Together, high‐mannose type *N‐*glycan is an enrichment marker for CSCs in human glioblastoma.

## 3 Discussion

We present evidence that the lower expression of MAN1A1 results in the formation of high‐mannose type *N‐*glycan of CD133 in GSCs. The interaction between high‐mannose CD133 and DNMT1 blocks the nuclear translocation of DNMT1. Activation of p21 and p27 expression by the CD133–DNMT1 interaction maintains GSC quiescence, self‐renewal, chemotherapy resistance, and tumorigenesis (Figure S8T, Supporting Information). Increasing evidence has shown that the quiescent state of CSCs protects them from DNA damage. The relatively quiescent state is necessary for preserving the self‐renewal of CSCs. The identification of the CD133/DNMT1 signaling axis provides a new insight into regulating the quiescence of GSC and a therapeutic target for the abrogation of quiescent CSCs.

### 3.1 The High Mannose Form of CD133 is Required for Its Interaction with DNMT1

The glycosylation status of CD133 is closely related to cell differentiation.^[^ ^22^ ^]^ We find that differentiation provokes CD133 glycosylation structure from high mannose type to complex type. The high‐mannose *N‐*glycan promotes the self‐renewal and tumorigenesis of GSCs by increasing the interaction between CD133 and DNMT1. High mannose *N‐*glycan promotes the metastasis, migration, and growth in vivo of cancer cells in various tissues.^[^ ^52^, ^55^ ^]^ Our findings provide a new role of high‐mannose *N‐*glycan in cancer progression.

The mechanisms by which the high‐mannose *N‐*glycan of CD133 regulates its interaction with DNMT1 remain unclear. Ectopic expression of MAN1A1 did not influence CD133 protein expression or delivery to the cell surface. Previous studies have shown that inhibition of the CD133 complex *N‐*glycosylation influences the recognition of the AC133 epitope without changing CD133 protein expression.^[^ ^56^ ^]^ *N‐*glycosylation regulates the structure of membrane protein.^[^ ^57^ ^]^ Thus, we presume that the *N‐*glycosylation of CD133 regulates its interaction with DNMT1 by influencing the CD133 structure on the cell surface.

### 3.2 High Mannose‐Type *N‐*Glycan is an Enrichment Marker for GSCs

Another important finding is that glioma cells with high mannose *N‐*glycan on cell surface showed characteristics consistent with CSCs. Actually, it has been reported that high mannose glycan on cell surface is associated with stem cell characteristic(s). For example, human embryonic stem cells were found to have high levels of high mannose glycan on cell surface.^[^ ^58^ ^]^ Glycosylation features associated with MSCs rather than differentiated cells included high‐mannose type *N‐*glycans.^[^ ^59^ ^]^ Accordingly, high mannose‐type *N‐*glycan is an enrichment marker for GSCs. Supporting this finding, high‐mannose glycans were increased according to HCC dedifferentiation.^[^ ^60^ ^]^ Growing evidence suggests that two distinct types of CSC lead to the formation of GBM. Type I CSC lines display “proneural” signature genes and are CD133 positive. Type II CSC lines show “mesenchymal” transcriptional profiles and lack CD133 expression.^[^ ^61^, ^62^ ^]^ Collectively, we presume that high‐mannose type *N‐*glycan might be an enrichment marker for proneural GSC.

### 3.3 Nuclear Localization of DNMT1 Inhibits the Slow‐Cycling State and Tumorigenesis of GSCs

The contributions of DNMT1 to CSCs have been extensively studied. In pancreatic ductal adenocarcinoma, pharmacologic or genetic targeting of DNMT1 in CSCs reduces their self‐renewal and in vivo tumorigenic potential.^[^ ^63^ ^]^ In breast cancer cells, DNMT1 deletion inhibited the self‐renewal and proliferation of cancer‐initiating cells.^[^ ^64^ ^]^ However, silencing DNMT1 promoted the induction of the CSC phenotype in prostate cancer cells.^[^ ^25^ ^]^ DNMT1 knockdown increases self‐renewal potential and tumorigenesis of hepatoma cells.^[^ ^26^ ^]^ In glioma, the level of 5‐mC is negatively related to the grade of glioma.^[^ ^65^ ^]^ Lower methionine inhibits the expression of DNMT1 and promotes the self‐renewal and tumorigenesis of GSCs.^[^ ^66^ ^]^ We found that nuclear DNMT1 promoted the proliferation of GSCs. The quiescent state of stem cells acts to limit the accumulation of DNA damage in normal and CSCs.^[^ ^35^ ^]^ Thus, nuclear DNMT1 inhibited the long‐term self‐renewal potential and tumorigenesis of GSCs. Collectively, DNMT1 has opposite effects on CSCs from different tumor sources. Glioblastoma is a rapidly evolving high‐grade astrocytoma by the presence of necrosis and microvascular hyperplasia.^[^ ^67^ ^]^ Quiescence or slow‐dying helps to maintain GSC in niche. Proneural GSCs expressed CD133 and mesenchymal GSCs lacked CD133 expression.^[^ ^62^ ^]^ Therefore, the CD133 mainly interacts with DNMT1 in the proneural GSC.

DNMT1 knockdown promotes the tumorigenesis of hepatoma stem cells through up‐regulation of BEX1.^[^ ^68^ ^]^ Here, we provided evidence that up‐regulation of p21 and p27 by CD133–DNMT1 interaction promotes the GSC quiescence. The quiescent or slow‐growing state of CSCs protects them from DNA damage. Accumulation of DNA damage results in the cell exhaustion.^[^ ^8^ ^]^ Thus, the quiescent or slow‐growing state of CSCs is necessary for the self‐renewal and tumorigenesis of CSC.^[^ ^7^ ^]^ Thus, we presume that DNA damage induced by the inhibition of CD133–DNMT1 interaction inhibited the self‐renewal and tumorigenesis of GSC. Our finding provides a new mechanism of GSC quiescence. However, CSCs display significant phenotypic and functional heterogeneity. For example, breast CSCs display plasticity transitioning between quiescent mesenchymal‐like (M) and proliferative epithelial‐like (E) states.^[^ ^69^ ^]^ The contribution of the CD133–DNMT1 interaction in the CSCs transition between quiescent and proliferative states needs further examination.

In summary, our results uncover CD133 as a crucial regulator of quiescence, self‐renewal, and tumorigenesis of GSCs. More importantly, elimination of the interaction between CD133 and DNMT1 by a cell‐penetrating peptide inhibits the self‐renewal and tumorigenesis of GSCs and increases the sensitivity of GSCs to temozolomide. Thus, targeting the interaction between CD133 and DNMT1 might help to abrogate quiescent CSCs. Our findings not only provide an improved understanding of the fundamental role of high‐mannose *N‐*glycan of CD133 in the tumorigenesis of GSCs, but also suggest an additional target for the abrogation of quiescent CSCs.

## 4 Experimental Section

### Isolation of CD133+ and CD133‐ Cells

CD133+ cells were isolated from primary surgical GBM biopsy specimens in accordance with protocols approved by the Fudan University Institutional Review Broads. All patients have been informed and consented to involve in this study. CD133+ and CD133‐ cells were isolated through magnetic cell sorting with CD133 cell isolation Kit (Miltenyi Biotec, cat#130‐100‐857) as previously described.^[^ ^2^ ^]^

### Isolation of Con A+ and Con A‐ Cells

Con A+ cells and Con A‐ were isolated from primary surgical GBM biopsy specimens. Fresh tissues were minced and treated with 0.2% collagenase (Sigma) and 1% Dispase II (Sigma) at 37 °C for 1–2 h. The resulting single‐cell suspensions were filtered through Cell Strainer (Corning). Cells were re‐suspended in MACS buffer containing biotinylated Con A antibody (Sigma) and incubated on ice for 30 min. After washing, the cells were incubated with Streptavidin‐conjugated MicroBeads for 15 min on ice. Positive cells were re‐suspended in MACS buffer containing biotinylated PHA‐L antibody and biotinylated DSL antibody (Sigma) and incubated on ice for 30 min. After washing, the cells were incubated with Streptavidin MicroBeads for 15 min on ice. PHA‐L and DSL‐positive cells were excluded. The ratio of high mannose *N‐*glycan in Con A+ cells and Con A‐ cells were then analyzed by FCS.

### Tumor Formation Assay

Intracranial transplantation of tumor cells into 6 to 8‐week old immunodeficient mice was performed in accordance with a Fudan University Institutional Animal Care and Use Committee‐approved protocol concurrent with national regulatory standards. Mice were maintained for up to 180 days or until the development of neurologic signs that significantly inhibited their quality‐of‐life (e.g., ataxia, lethargy, seizures, inability to feed, etc.).

To examine the effect of peptides on the growth of GSCs, cells treated with the peptides were subcutaneously injected into immunodeficient mice. Tumor size was measured and their volumes were calculated using the equation of *L* (length) × *W* (width)^2^/2.

### Neurosphere Formation Assay

For single‐cell neurosphere formation assay, 48 h after treatment with the indicated lentivirus, cells were trypsinized and single‐cell suspensions were cultured in 24‐well plates containing supplemented DMEM/F12 medium with lower concentration growth factor (2 ng mL^−1^ EGF). After 5 days, the number of neurospheres/well was quantified (passage 1). For secondary/tertiary neurospheres formation assay (passages 2 and 3), the established neurospheres were dissociated into single cells and were cultured in 24‐well plates. The number of spheres with secondary neurospheres was counted after 5 days.

For the limiting dilution assay of GSCs, 0, 5, 10, 15, 20, and 25 cells were seeded into a 24 well plate each. After 2 weeks, the number of wells which had neurospheres was counted (*n* = 10), ***p < 0.001 by ELDA analysis.^[^ ^70^ ^]^

### Analysis of GBM Subtype

Briefly, RNA from patient samples was extracted and profiled on Affymetrix Human Exon 1.0 ST Gene Chips according to the manufacturer's protocol. Proneural, neural, classical, and mesenchymal GBM subtypes were determined by clustering of expression data from the Affymetrix HuEx array platform using the previously published gene marker.^[^ ^71^ ^]^

### Cell Cultures

The sorted CD133+ cells were cultured in the DMEM/F12 media supplemented with B27 lacking vitamin A (Invitrogen), 2 µg mL^−1^ heparin (Sigma), 20 ng mL^−1^ EGF (Chemicon), and 20 ng mL^−1^ FGF‐2 (Chemicon) for a short period before treatment and analysis. CD133^−^ tumor cells were plated in DMEM with 10% fetal bovine serum for at least 12 h to permit cell survival. Prior to performing experiments with CD133^–^ cells, DMEM with 10% fetal bovine serum was replaced with supplemented DMEM/F12 media in order for experiments to be performed in identical media.

### Western Analysis

Equal amounts of cell lysate were resolved by SDS‐PAGE, transferred to polyvinylidene difluoride (PVDF) membranes (Roche). Blocking was performed for 60 min with 5% nonfat dry milk or %1 BSA in TBST and blotting was performed with primary antibodies for 12–16 h at 4 °C. Primary antibodies included: mouse monoclonal anti‐CD133 (W6B3C1 clone) (Miltenyi Biotec, cat# 130‐092‐395; 1:1000), rabbit polyclonal anti‐FLAG (Sigma, cat# F7425; 1:3000), mouse monoclonal anti‐DNMT1 (Abcam, cat# ab13537, 1:2000), rabbit polyclonal anti‐DNMT2 (Abcam, cat# ab82659, 1:2000), goat polyclonal anti‐DNMT3a (R&D, cat# AF6315, 1:1000), rabbit monoclonal anti‐p21 (Cell signaling, CST#2947, 1:1000).

After extensive washing with TBST, the membranes were incubated for 1.5–2 h at room temperature (RT) with HRP‐conjugated goat anti‐rabbit antibody (Santa Cruz Biotechnology, cat# sc‐2004; 1:3000), goat anti‐mouse antibody (Santa Cruz Biotechnology, cat# sc‐2031; 1:2000) or rabbit anti‐goat secondary antibody (Sigma, cat# A5420; 1:50000), and signal was detected by enhanced chemiluminescence substrate (Pierce Biotechnology). For quantification, the western blot films were scanned and were densitometrically analyzed using ImageJ Version 1.33u software.

### Immunofluorescence

For immunostaining of undifferentiated tumor spheres, cells were fixed with 4% PFA for 20 min at RT, washed three times with PBS, and then blocked with a PBS‐based solution containing 5% normal serum and 0.3% Triton X‐100. Cells were incubated overnight at 4 °C with mouse monoclonal anti‐Nestin (Millipore, cat# MAB5326; 1:200). After washed three times with PBS, cells were incubated with Alexa 488 conjugated goat anti‐mouse IgG (Invitrogen, 1:400). Nuclei were counterstained with DAPI or Hoechst 33258 (Sigma; 10 µg mL^−1^).

For examining the differentiation capacity of GSC, CD133^+^ tumor cells were plated onto poly‐lysine‐coated coverslips in DMEM containing 2% fetal bovine serum for 7 days. After cells had attached, spread out, and underwent distinct morphological changes, they were fixed, blocked, and incubated overnight at 4 °C with the appropriate antibody: mouse monoclonal anti‐Map2 (Sigma, cat# M4403; 1:200), rabbit polyclonal anti‐GFAP (Millipore, cat# AB5804; 1:250), or mouse monoclonal anti‐O4 (IgM) (Sigma, cat# O7139; 1:200). Cells were washed three times with PBS and incubated with the appropriate secondary antibody: Alexa 594‐conjugated goat anti‐mouse IgG (Invitrogen; 1:400), Alexa 488‐conjugated goat anti‐rabbit IgG (Invitrogen; 1:400), or Alexa 488‐conjugated goat anti‐mouse IgM (Invitrogen; 1:400). Nuclei were counterstained with DAPI or Hoechst 33258 (Sigma; 10 µg mL^−1^).

For immunostaining analysis of endogenous CD133 and DNMT1 co‐localization, CD133+ cells were fixed with 4% PFA for 20 min at RT, washed three times with PBS, and then blocked with a PBS‐based solution containing 5% normal goat serum and 0.3% Triton X‐100. Cells were co‐incubated overnight at 4 °C with rabbit polyclonal anti‐DNMT1 antibody (Cell signaling, cat# 5032; 1:100) and mouse monoclonal anti‐CD133 (W6B3C1 clone) (Miltenyi Biotec, cat# 130‐092‐395; 1:50). After washed three times with PBS, cells were co‐incubated with Alexa 488‐conjugated goat anti‐mouse IgG (Invitrogen; 1:400) and Alexa 594‐conjugated goat anti‐rabbit IgG (Invitrogen, 1:400). Nuclei were counterstained with DAPI (Sigma). Immunofluorescent images were collected on a Leica TCS SP5 confocal microscope and analyzed using LAS AF software.

To analysis of the interaction between exogenous CD133‐ GFP and DNMT1‐dsRed, CD133+ cells were cultured in poly‐L‐lysine/laminin‐coated plates as previously described.^[^ ^72^ ^]^ CD133^+^ cells were fixed with 4% PFA for 20 min at RT, washed three times with PBS, and then blocked with a PBS‐based solution containing 5% normal goat serum and 0.3% Triton X‐100. Cells were co‐incubated overnight at 4 °C with mouse anti‐GFP antibody (Roche, cat# 11814460001; 1:100) and rabbit anti‐dsRed (Clontech, cat# 632496; 1:100). After washed three times with PBS, cells were co‐incubated with Alexa 488‐conjugated goat anti‐mouse IgG (Invitrogen; 1:400) and Alexa 594‐conjugated goat anti‐rabbit IgG (Invitrogen, 1:400). Nuclei were counterstained with DAPI or Hoechst (Sigma). Immunofluorescent images were collected on a Leica TCS SP5 confocal microscope and analyzed using LAS AF software.

### Subcellular Fractionation

Nuclear fractions, used for the assessment of DNMT1 nuclear expression, were extracted with Subcellular Protein Fractionation Kit (Pierce Biotechnology, cat# 78840) following manufacturer's instruction. Equal amounts of nuclear fractions were resolved by SDS‐PAGE, and analyzed by western blotting using the indicated antibody. Antibodies included: Histone H3 (Cell Signaling, cat# 4499; 1:1000), *α*‐Tubulin (Sigma, cat# T5168, 1:1000) and DNMT1 (Abcam, cat# ab13537, 1:2000). Histone H3 was used as the nuclear marker, and *α*‐Tubulin was used as the cytoplasmic marker.

### Plasmids

To knockdown endogenous CD133 expression, the CD133 shRNA lentivirus vectors were generated by ligation of lentivirus vector pLL3.7 containing contains neomycin gene with oligonucleotides (5’‐TGGCTTGGAATTATGAATTGTTCAAGAGACAATTCATAATTCCAAGCCTTTTTTC‐3′) or (5′‐ TGCTCAGAACTTCATCACAATTCAAGAGATTGTGATGAAGTTCTGAGCTTTTTTC‐3′) (underlines indicate the target sequence for CD133 shRNA1 or CD133 shRNA2). Control shRNA lentivirus vector utilized for experimental control was generated by ligation of pLL3.7 vector with oligonucleotides (5′‐ TGTGACCAGCGAATACCTGTTTCAAGAGAACAGGTATTCGCTGGTCACTTTTTTC‐3′) (underline indicates the target sequence for Control shRNA).

For ectopic expression of CD133, the LV‐CD133‐ FLAG plasmid was constructed by inserting full‐length human CD133 cDNA into the LV‐FLAG lentivirus vector between BamHI and AgeI sites. CD133 deletion mutants (CD133(1–862), CD133(1–857), CD133(1–840), CD133(1–835), CD133(1–824)) were created using Takara MutanBEST mutagenesis kit. Mutated constructs were sequenced, and the correct ones were selected for further experiments. FLAG‐DNMT1 Del(155‐163) C1229S was construed from FLAG‐linker‐DNMT1 Del (155‐163) using Takara mutagenesis kit. The position of catalytic cysteine in the FLAG‐DNMT1Del (155‐163) protein was 1229. To generate shRNA‐resistant CD133 (shR‐CD133‐FLAG) or shRNA‐resistant CD133(1–862)‐FLAG (shR‐CD133(1–862)‐FLAG) lentivirus vectors, site‐directed mutagenesis technique was used to introduce four mutations into the coding region of CD133 or its deletion mutants (nucleotides 90–108) cognate to the CD133 shRNA1 target sequence (GGC*A*TGGAA*C*TA*C*GA*G*TT*A*, mutations italicized), and the introduction of these mutations was confirmed by sequencing.

For ectopic expression of p27 or p21, the LV‐P21‐FLAG or LV‐P27‐FLAG plasmid was constructed by inserting full‐length human p21 cDNA or p27 cDNA into the LV‐FLAG lentivirus vector between BamHI and AgeI site.

### Immunoprecipitation (IP)

GBM tissues or cells were lysated in a modified RIPA buffer (50 mM Tris (pH 7.4), 1% Triton X‐100, 150 mM NaCl, 2 mM EDTA, protease inhibitor cocktail, 1 mM *β*‐glycerophosphate, 1 mM Na_3_VO_4_, 1 mM NaF). The lysates were centrifuged and cleared by incubation with 25 µl of Protein G‐Agarose (Roche) for 1.5 h at 4 °C. The pre‐cleared supernatant was subjected to IP using the indicated first antibodies at 4 °C overnight. Antibodies used in IP included: mouse monoclonal anti‐CD133 (W6B3C1 clone) (Miltenyi Biotec, cat# 130‐092‐395) and mouse monoclonal anti‐DNMT1 (Abcam, cat# ab13537). Then, the protein complexes were collected by incubation with 30 µl of Protein G‐Agarose (Roche) for 2 h at 4 °C. The collected protein complexes were washed four times with IP buffer and analyzed by western blotting using indicated antibodies. Antibodies included: rabbit polyclonal anti‐DNMT1 antibody (Abcam, cat# ab13537), mouse monoclonal anti‐CD133 (W6B3C1 clone) (Miltenyi Biotec, cat# 130‐092‐395; 1:1000).

### Strep Pull‐Down Assay

2 µg Strep (Trp‐Ser‐His‐Pro‐Gln‐Phe‐Glu‐Lys) or Strep‐CD133(813–865) (CD133 C‐terminal cytoplasmic domain; amino acids 813–865) protein purified from bacteria BL21 bound to Tactin agarose beads or CD133‐strep protein purified from 293T cells were incubated with recombinant DNMT1 (Active motif, cat# 31 404) in binding buffer (20 mM Hepes‐KOH (pH 7.6), 2.5 mM MgCl_2_, 200 mM KCl, 0.5 mM EDTA, 1 mM DTT, 0.1% NP‐40, 10% Glycerol, 1 mM PMSF) at 4 °C for 6 h. After washing with binding buffer, the pull down products were subjected to SDS‐PAGE, and were analyzed by western blotting using the indicated antibody. Antibodies included: rabbit polyclonal anti‐DNMT1 antibody (Abcam, cat# ab13537), HRP conjugated monoclonal mouse anti‐ Strep•Tag II antibody (Millipore, cat#71591‐3).

### DNA methylation analysis

Cytosine methylation was determined using bisulfite sequencing. Briefly, genomic DNA was subjected to bisulfite conversion with the EpiTect bisulfite kit (Qiagen) according to the manufacturer's instructions. Modified DNA was purified with a Qiagen Gel Extraction Kit. The fragments, which encompasses the CpG sequences in this region of p21 and p27 promoters was amplified by PCR using modified DNA as templates. The PCR products were sub‐cloned into a TA‐cloning vector. 50 clones for each sample was sequenced. The amount of mC relative to global cytidine (5 mC + dC) can be calculated for each sample, and this can be compared between the experimental and control samples.

### Global DNA methylation quantification

The global DNA methylation (5‐mC) was quantified by MethylFlash Methylated DNA Quantification Kit (Epigentek). Briefly, 50 ng of DNA from CD133+ cells and CD133‐ cells was used for incubation with both capture and detection antibodies. After washed with PBS for at least three times, the absorbance of the sample was measured in a microplate spectrophotometer at 450 nm (BioTek Instruments, USA). The percentage of the whole genome 5‐methylcytosine (5‐mC) was calculated according to manufacturer's instructions.

### Cell cycle analysis

CD133+ cells expressing the indicated plasmids were rinsed with phosphate buffered saline (pH 7.4) and then collected by centrifugation at 4 °C. Pellets were re‐suspended in ice cold 70% ethanol and rinsed in PBS for three times. Then, cells were re‐suspended in PBS containing 20 µg mL^−1^ propidium iodide (PI). After washed for three times, Fluorescence was measured using a flow cytometer.

### DNA methylation microarray

DNA methylation microarray analysis of human cells was analyzed using the Infinium® MethylationEPIC BeadChip(Illumina, San Diego, CA)according to Illumina's instruction. DNA methylation levels (*β* values) of individual genomic blocks were evaluated using the mean *β* values of all the probes within individual genomic blocks. Initially, probes containing single nucleotide polymorphisms was excluded. Next, the K‐nearest neighbor method was used and the *β* mixture quantile amplification method for imputation and normalization, respectively. Subsequently, the threshold value 0.1 of average *β* in each group was filtered, and the P value based on the false discovery rate was 0.001.

### Chromatin Immunoprecipitation

Analysis the binding of DNMT1 to p21 promoter region was performed using chromatin immunoprecipitation (ChIP) assay kit (Upstate, 17–295). Briefly, cells were cross‐linked by addition of 1% formaldehyde for 15 min at 37 °C. After washed with cold PBS, cells were lysed in an SDS lysis buffer (1% SDS, 50 mM Tris at pH 8, 20 mM EDTA). The lysates were sonicated to shear DNA to lengths between 150 and 700 base pairs (bp). After tenfold dilution in ChIP dilution buffer (16.7 mM Tris, 0.01% SDS, 1.1% Triton X‐100, 1.2 mM EDTA, 167 mM NaCl), IPs were carried out overnight at 4 °C with 2 µg of DNMT1 (Abcam, cat# ab13537) or 2 µg of normal mouse IgG as a negative control. Fifty microliters of protein G beads were added to each sample for 4 h, and the beads were then washed as per the Upstate Biotechnology ChIP protocol. DNA was eluted twice with 100 µL of TE with 1% SDS for 10 min at 65 °C. The cross‐links were reversed overnight at 65 °C. Proteinase K was added for 1 h at 65 °C, and then DNA was recovered by phenol extraction and ethanol precipitation. Immunoprecipitated DNA was analyzed for the presence of the p21 promoter by PCR.

### Lectin Blot

The immunoprecipitates were subjected to western blot analysis according to the standard procedures. For lectin staining, the PVDF membrane was blocked in 5% BSA in TBS for 4 h at RT. The membrane was washed twice for 10 min with TBS, then once with lectin vehicle (1 mM MgCl_2_, 1 mM MnCl_2_, and 1 mM CaCl_2_ in TBS) before 1 h incubation with biotinylated lectin (1:2000, Vector Laboratories). The membrane was washed three times for 10 min each in TBST (1% Tween‐20 in TBS), and were then incubated 45 min with Streptavidin‐HRP (1:2000, Southern Biotech).

### Yeast Two‐Hybrid Analysis

The cDNA encoding C‐terminal cytoplasmic domain of CD133 (residues 813–865) was cloned into pGBKT7 vector and was used as the bait to screen the pACT2‐human cDNA libraries (human fetal brain). Positive interactions were verified by *β*‐galactosidase assay.

### Purification of Strep‐Tagged CD133 Protein

HEK293T cells expressing CD133 and its mutant were treated with *N‐*glycosylation inhibitor Kifunensine. Cells were lysed at 4 °C for 2 h using lysis buffer (150 mM NaCl, 100 mM Tris (pH 8.0), 0.5% Triton X‐100, 1 mM EDTA, protease inhibitor mixture). The supernatants of cell lysates were incubated with Strep‐Tactin agarose at 4 °C for 14–18 h. After incubation, the agarose was washed three times in lysis buffer containing 2 M NaCl to eliminate nonspecific proteins. Desthiobiotin (2.5 mM) was used to elute Strep‐CD133 proteins. The elution of CD133 protein was concentrated using an ultrafiltration tube. The purified effect of CD133 protein was determined by Coomassie Blue staining.

Measurement of DNMT1 binding to CD133 and its mutants by ELISA. ELISA was performed as previously described.^[^ ^73^ ^]^ ELISA plate wells were coated with a CD133 antibody (Miltenyi Biotec, Cat # 130‐092‐395) by incubating 1 µg/100 µL of the antibody per well at 4 °C for 12–14 h. After wells were washed with PBST (PBS with 0.05% Tween 20), wells were blocked by incubation with PBS containing 1% bovine serum albumin (BSA) for 2 h at RT. Next, each well was added with serially diluted recombinant human DNMT1‐his (Active motif) (final concentration: 0.01, 0.1, 1, 10, 40, 80, 160, 640, 1280, and 2560 nM). Purified strep‐tagged human CD133 or CD133 mutant (final concentration: 250 nM) were added to each DNMT1‐containing well. After 2 h at 37 °C, each well was washed with PBST and incubated with anti‐DNMT1 antibody (abcam) for 2 h at RT. After being washed with PBST for 3 times, the wells were examined by horseradish peroxidase‐based detection systems. After adequate color development, 100 µl per well of STOP solution was added, followed by absorbance reading at 450 nm by the Microplate Reader from BioTek.

### Statistical Analysis

In general, significance was tested by unpaired two‐tailed Student's *t* test using GraphPad InStat 5.0 software. For animals’ studies, Kaplan Meier curves and log‐rank analysis were performed. *P* values < 0.05 were considered statistically significant, with *p < 0.05, **p < 0.01, ***p < 0.001, respectively. Results are expressed as the mean ± standard deviation (SD) from at least 3 independent experiments.

## Conflict of Interest

The authors declare no conflict of interest.

## Author Contributions

Y.W., Q.C., S.H., and Y.L. contributed equally to this work. Y.W., Q.C., S.H., and Y.L. conducted experiments, performed the experiments, and collected data. Y.L., Z.J., W.L., Y.X., D.S., B.W., and X.C. performed the experiments. Y.L. and W.X. provided patients’ samples. Y.W. and J.J. wrote manuscript. J.J. designed experiments and wrote manuscript. All authors approved the final version of the manuscript.

## Supporting information

Supporting Information

## Data Availability

The data that support the findings of this study are available in the supplementary material of this article.
