# Supplementary material for: The Interaction between DNMT1 and High‐Mannose CD133 Maintains the Slow‐Cycling State and Tumorigenic Potential of Glioma Stem Cell
Source: Adv Sci (Weinh). 2022 Jul 7;9(26):2202216. doi: 10.1002/advs.202202216 (PMC9475542; doi:10.1002/advs.202202216)
Supplement: Supplementary file 1 — Supporting Information [file ADVS-9-2202216-s001.pdf]

ADVANCED SCIENCE
Open Access

Supporting Information

for *Adv. Sci.*, DOI 10.1002/advs.202202216

# The Interaction between DNMT1 and High-Mannose CD133 Maintains the Slow-Cycling State and Tumorigenic Potential of Glioma Stem Cell

*Yuanyan Wei, Qihang Chen, Sijing Huang, Yingchao Liu, Yinan Li, Yang Xing, Danfang Shi, Wenlong Xu, Weitao Liu, Zhi Ji, Bingrui Wu, Xiaoning Chen and Jianhai Jiang**

**The Interaction between DNMT1 and High-Mannose CD133 Maintains the Slow-Cycling State and Tumorigenic Potential of Glioma Stem Cell**

**Yuanyan Wei[1#], Qihang Chen[1#], Sijing Huang[1#], Yinchao Liu[2#], Yinan Li[1], Yang Xing[1], Danfang Shi[1], Wenlong Xu[3], Weitao Liu[1], Zhi Ji[1], Bingrui Wu[1], Xiaoning Chen[1], Jianhai Jiang[1]***

## Supplemental Information Inventory

**Figure S1.** Characterization of CD133+ and CD133− cells from human GBM specimens, related to Figure 1

**Figure S2.** The CD133-DNMT1 interaction inhibits the nuclear translocation of DNMT1, related to Figure 2

**Figure S3.** The CD133-DNMT1 interaction up-regulates p21 and p27, related to Figure 3

**Figure S4.** The nuclear localization of DNMT1 inhibits the self-renewal ability and tumorigenesis of glioma stem cells, related to Figure 4

**Figure S5.** The CD133-DNMT1 interaction maintains the self-renewal capacity of glioma stem cell, related to Figure 5

**Figure S6.** The effect of the interaction between CD133 and DNMT1 on the DNA damage in glioma stem cell, related to Figure 6

**Figure S7.** The effect of the interaction between CD133 and DNMT1 on the apoptosis of glioma stem cell induced by TMZ, related to Figure 7

**Figure S8.** The high-mannose N-glycan of CD133 is necessary for its interaction with DNMT1, related to Figure 8

**Table S1.** Screening protein interacting with CD133 c-terminal (aa813-865) using the yeast two hybrid system, related to Figure 1

**Table S2.** Pathologic and cytogenetic characteristics of brain tumors, related to Figure 1

**Table S3.** Gene ontology results for 680 genes which methylation is upregulated in CD133+ cells expressing FLAG-DNMT1(Del(155-163)) were shown ($p$ < 0.05), related to Figure 3.

**Table S4.** The methylation of genes inhibiting cell cycle progression are upregulated CD133+ cells expressing DNMT1 (Del(155-163)) ($p$ < 0.001), related to Figure 3.

# Figure S1

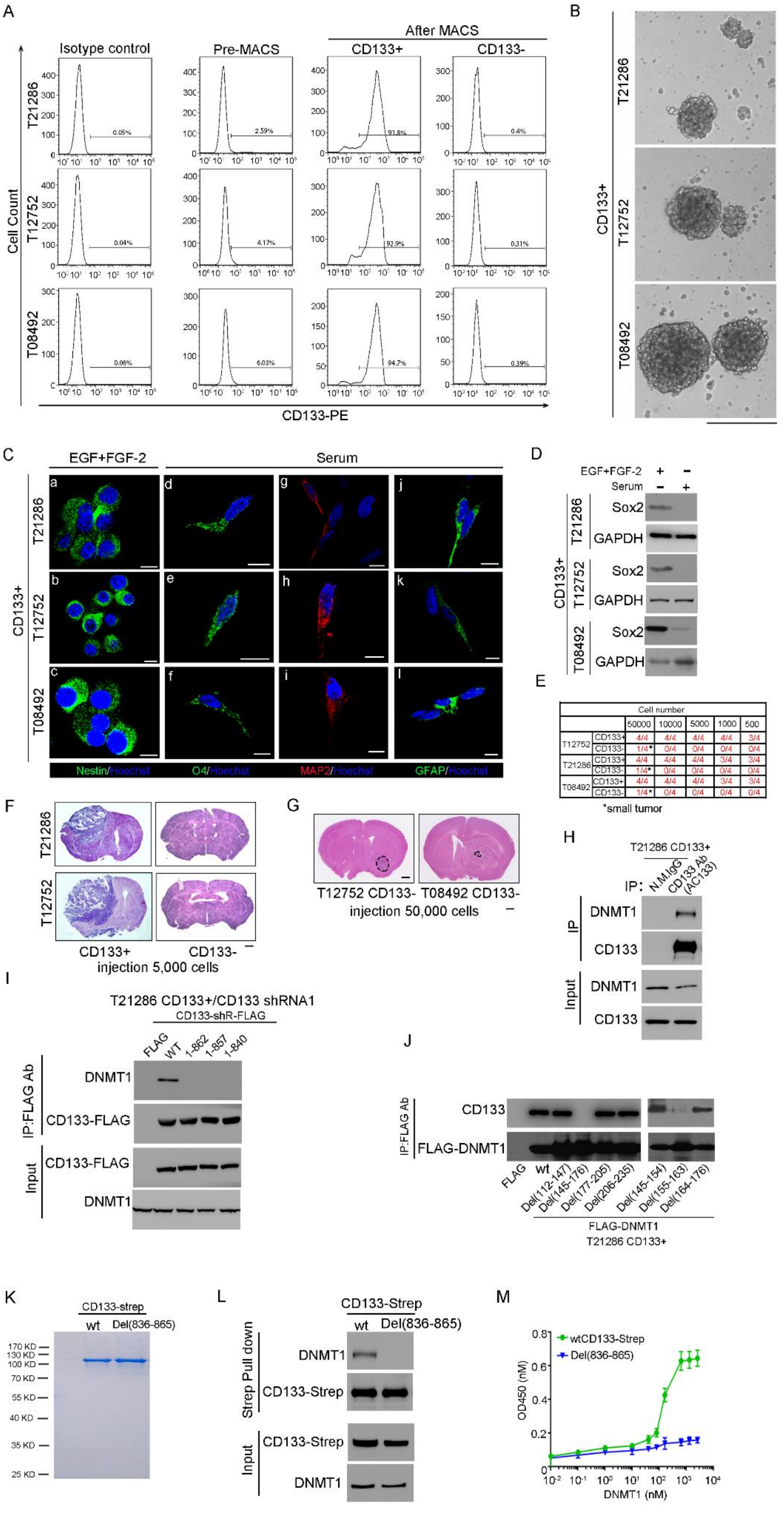


| Cell number | | | | | | |
|---|---|---|---|---|---|---|
| | | 50000 | 10000 | 5000 | 1000 | 500 |
| T12752 | CD133+ | 4/4 | 4/4 | 4/4 | 4/4 | 3/4 |
| | CD133- | 1/4* | 0/4 | 0/4 | 0/4 | 0/4 |
| T21286 | CD133+ | 4/4 | 4/4 | 4/4 | 3/4 | 3/4 |
| | CD133- | 1/4* | 0/4 | 0/4 | 0/4 | 0/4 |
| T08492 | CD133+ | 4/4 | 4/4 | 4/4 | 3/4 | 3/4 |
| | CD133- | 1/4* | 0/4 | 0/4 | 0/4 | 0/4 |

*small tumor

**Figure S1. Characterization of CD133+ and CD133− cells from human GBM specimens, related to Figure 1**

A. Fluorescence-activated cell sorting (FACS) analysis of CD133 expression before and after sorting of CD133+ and CD133− fractions. CD133+ cells were isolated from freshly dissociated GBM samples (T21286, T12752, and T08492) by magnetic beads. The percentage of CD133-positive cells was determined by FACS analysis relative to cells labeled with a fluorophore-conjugated IgG isotype control antibody.

B. Representative image of neurospheres derived from CD133+ cells isolated from glioblastoma specimens (T21286, T12752, and T08492). Scale bar represents 100 μM.

C. (a-c) CD133+ cells expressed the stem cell marker Nestin (green), as assessed by immunofluorescence. (d-l) Multilineage differentiation capacity of CD133+ cell was evaluated by staining for the O4 oligodendrocytic marker (d-f, green), the MAP2 neuron marker (g-i, red) and the GFAP astrocyte marker (j-l, green). Nuclei were counterstained with Hoechst 33258. "EGF+FGF-2" indicates the supplemented DMEM/F12 condition. "Serum" indicates the addition of serum. Scale bars represent 10 μM.

D. Western blot analysis of the protein level of Sox2 in CD133+ cells treated with or without 2% FBS for 7 days. GAPDH was used as a loading control.

E-G. An in vivo limiting dilution tumor formation assay (employing 50,000, 10,000, 5,000, 1,000 or 500 cells per mouse) was performed to compare the tumor-initiating capacity of CD133+ glioma cells with matched CD133- glioma cells. Mice were sacrificed when they were moribund or 180 days after implantation. Tumor formation was determined by histology. E. The table displays the number of mice developing tumors. F. H&E staining of mouse brain shows tumors formation by 5,000 CD133+ but not by 5000 CD133- cells. G. H&E staining of mouse brain showed occasional small tumors formation by $5\times10^5$ CD133- cells. Scale bar, 1 cM.

H. CD133 interacts with DNMT1 in vivo. Lysates of CD133+ cells isolated from glioblastoma samples were subjected to IP using anti-CD133 (clone AC133) Ab, followed by IB with anti-CD133 Ab or anti-DNMT1 Ab. Whole-cell lysates were analyzed by IB with anti-CD133 or anti-DNMT1 antibodies as input.

I. Co-IP assay was performed to determine which region of CD133 was essential for its interaction with DNMT1. The lysates of CD133+ cells expressing CD133 shRNA1 and shRNA-resistant c-terminal deletion mutant with FLAG tag were subjected to IP

using anti-FLAG antibody, followed by IB with anti-FLAG or anti-DNMT1 antibodies. shR: shRNA-resistant.

J. Co-IP analysis was performed to determine the region of DNMT1 which was essential for its interaction with CD133. The lysates of CD133+ cells expressing FLAG or DNMT1-FLAG or DNMT1 deletion mutants were subjected to IP using anti-FLAG Ab, followed by IB with anti-FLAG Ab or anti-CD133 Ab.

K. CD133 protein with Strep tag was purified by Strep-Tactin affinity from 293T cells treated with N-glycosylation inhibitor Kifunensine. CD133 protein was eluted with 2 M NaCl in wash buffer (100 mM Tris-HCl, 1 mM EDTA, pH 8.0) to eliminate nonspecific proteins. By Coomassie brilliant blue staining, the purity of CD133 purified protein was over 90%.

L. In vitro interaction between full length CD133-Strep and purified DNMT1. Purified CD133-Strep proteins or its mutant were incubated with purified DNMT1 protein. The Strep pull down were blotted with anti-Strep and anti-DNMT1 antibodies.

M. Human DNMT1 protein (10–2560 nM) was incubated with purified CD133-strep or CD133 mutant Del(848-865) (250 nM) overnight at 4 °C. Their binding was measured by ELISA. Results are expressed as mean ± SD from three independent experiments.

Figure S2

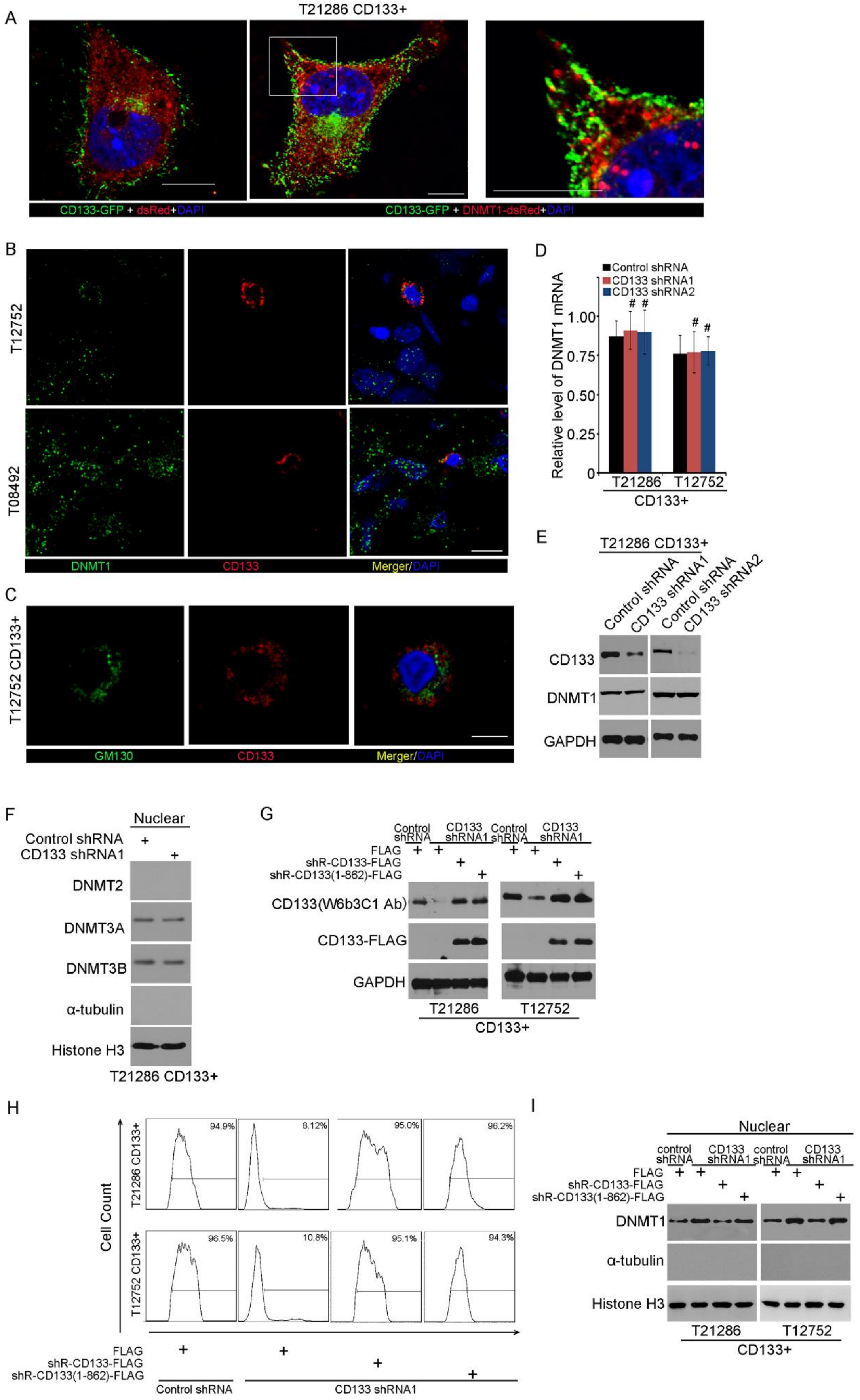
A
T21286 CD133+
CD133-GFP + dsRed+DAPI
CD133-GFP + DNMT1-dsRed+DAPI
B
T12752
T08492
DNMT1
CD133
Merger/DAPI
C
T12752 CD133+
GM130
CD133
Merger/DAPI
D
Control shRNA
CD133 shRNA1
CD133 shRNA2
Relative level of DNMT1 mRNA
1.00
0.75
0.50
0.25
0
T21286
T12752
CD133+
E
T21286 CD133+
Control shRNA
CD133 shRNA1
Control shRNA
CD133 shRNA2
CD133
DNMT1
GAPDH
F
Nuclear
Control shRNA
CD133 shRNA1
DNMT2
DNMT3A
DNMT3B
α-tubulin
Histone H3
T21286 CD133+
G
Control shRNA
CD133 shRNA1
FLAG
shR-CD133-FLAG
shR-CD133(1-862)-FLAG
CD133(W6b3C1 Ab)
CD133-FLAG
GAPDH
T21286
T12752
CD133+
H
Cell Count
T21286 CD133+
T12752 CD133+
94.9%
8.12%
95.0%
96.2%
96.5%
10.8%
95.1%
94.3%
FLAG
shR-CD133-FLAG
shR-CD133(1-862)-FLAG
Control shRNA
CD133 shRNA1
I
Nuclear
control shRNA
CD133 shRNA1
DNMT1
α-tubulin
Histone H3
T21286
T12752
CD133+

**Figure S2. The CD133-DNMT1 interaction inhibits the nuclear translocation of DNMT1, related to Figure 2**

A. Co-localization of CD133-GFP and deRed (right panel) or DNMT1-dsRed (middle panel and left panel) was assessed by immunofluorescence staining of CD133 (GFP) and DNMT1 (dsRed) in T21286 CD133+.cells expressing CD133-GFP and deRed or DNMT1-dsRed cultured in poly-L-lysine/laminin-coated plates. Nuclei (blue) were counterstained with DAPI. Co-localization of CD133 and DNMT1 is demonstrated by yellow fluorescence. Scale bars, 10 μM.

B. Co-localization of CD133 and DNMT1 was assessed by immunofluorescence staining of CD133 (red) and DNMT1 (green) in glioblastoma tissues T12752 (upper panel) and T08492 (lower panel). Nuclei (blue) were counterstained with DAPI. Co-localization of CD133 and DNMT1 is demonstrated by yellow fluorescence. Scale bars, 10 μM.

C. Co-localization of CD133 and Golgi 130 was assessed by immunofluorescence staining in CD133+ cells. Nuclei (blue) were counterstained with DAPI. Scale bars, 10 μM.

D. qRT-PCR quantification of the mRNA level of DNMT1 in CD133+ cells expressing control shRNA, CD133 shRNA1 or CD133 shRNA2. Results are expressed as mean ± SD from three independent experiments; #, ns. Student's t-test.

E. Western blot analysis of the protein level of DNMT1 or CD133 in CD133+ cells expressing control shRNA, CD133 shRNA1 or CD133 shRNA2. GAPDH was used as a loading control.

F. The level of nuclear DNMT2, DNMT3A and DNMT3B in CD133+ cells expressing control shRNA or CD133 shRNA1 was determined by immunoblotting. Histone H3 was used as the nuclear marker, and α-tubulin was used as the cytosolic marker.

G. The protein level of CD133 in CD133+ cells expressing control shRNA, CD133 shRNA1, CD133 shRNA1+shRNA-resistant wild-type CD133-FLAG, or CD133 shRNA1+shRNA-resistant CD133(1-862)-FLAG was determined by immunoblotting with CD133 Ab and FLAG Ab. GAPDH was used as a loading control.

H. FCS analysis of cell surface CD133 expression in CD133+ cells expressing control shRNA, CD133 shRNA1, CD133 shRNA1+shRNA-resistant wild-type CD133-FLAG, or CD133 shRNA1+shRNA-resistant CD133(1-862)-FLAG.

I. The level of nuclear DNMT1 in CD133+ cells expressing control shRNA, CD133

shRNA1, CD133 shRNA1 + shRNA-resistant wild-type CD133, or CD133 shRNA1 + shRNA-resistant CD133(1-862) mutant was determined by immunoblotting. Histone H3 was used as the nuclear marker, and α-tubulin was used as the cytosolic marker. The figures are presented out of three independent experiments.

Figure S3

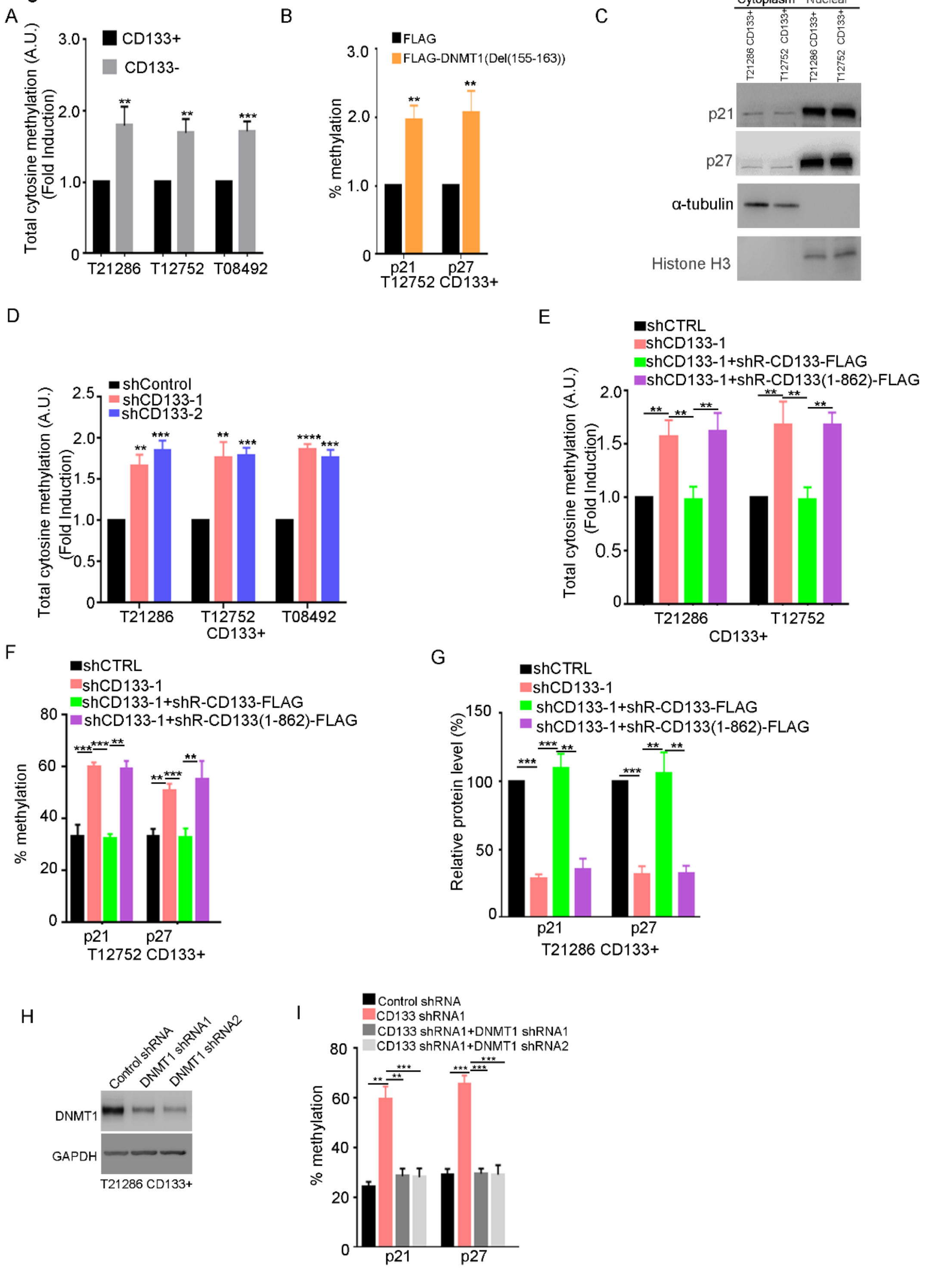
A
CD133+
CD133-
Total cytosine methylation (A.U.)
(Fold Induction)
T21286 T12752 T08492
B
FLAG
FLAG-DNMT1(Del(155-163))
% methylation
p21 p27
T12752 CD133+
C
Cytoplasm Nuclear
T21286 CD133+
T12752 CD133+
p21
p27
α-tubulin
Histone H3
D
shControl
shCD133-1
shCD133-2
Total cytosine methylation (A.U.)
(Fold Induction)
T21286 T12752 T08492
CD133+
E
shCTRL
shCD133-1
shCD133-1+shR-CD133-FLAG
shCD133-1+shR-CD133(1-862)-FLAG
Total cytosine methylation (A.U.)
(Fold Induction)
T21286 T12752
CD133+
F
shCTRL
shCD133-1
shCD133-1+shR-CD133-FLAG
shCD133-1+shR-CD133(1-862)-FLAG
% methylation
p21 p27
T12752 CD133+
G
shCTRL
shCD133-1
shCD133-1+shR-CD133-FLAG
shCD133-1+shR-CD133(1-862)-FLAG
Relative protein level (%)
p21 p27
T21286 CD133+
H
Control shRNA
DNMT1 shRNA1
DNMT1 shRNA2
DNMT1
GAPDH
T21286 CD133+
I
Control shRNA
CD133 shRNA1
CD133 shRNA1+DNMT1 shRNA1
CD133 shRNA1+DNMT1 shRNA2
% methylation
p21 p27

**Figure S3. The CD133-DNMT1 interaction up-regulates p21 and p27, related to Figure 3**

A. The level of total 5-methylcytosine in CD133+ cells and CD133- cells was examine by ELISA kit. Values are normalized to that of CD133+ cells. Results are expressed as mean ± SD from three independent experiments; $***p < 0.001$, $**p < 0.01$, Student's t-test.

B. The methylation ratio of p21 and p27 promoter in CD133+ cells expressing control or FLAG-DNMT1(Del(155-163)). Methylation levels are determined by bisulfite sequence. Results are expressed as mean ± SD from three independent experiments; $**p < 0.01$, Student's t-test.

C. Western blot was performed to analyze the level of nuclear p21 and nuclear p27 in CD133+ cells. Histone H3 was used as the nuclear marker, and α-tubulin was used as the cytosolic marker.

D. The level of total 5-methylcytosine in CD133+ cells expressing control shRNA, CD133 shRNA-1 or CD133 shRNA-2 was examine by ELISA kit. Values are normalized to that of CD133+ cells. Results are expressed as mean ± SD from three independent experiments; $***p < 0.001$, $**p < 0.01$, Student's t-test.

E. The level of total 5-methylcytosine in CD133+ cells expressing control shRNA, CD133 shRNA1, CD133 shRNA1+shRNA-resistant wild-type CD133, or CD133 shRNA1+shRNA-resistant CD133(1-862) mutant. was examine by ELISA kit. Values are normalized to that of CD133+ cells. Results are expressed as mean ± SD from three independent experiments; $**p < 0.01$, Student's t-test.

F. Bisulfite sequence assay was performed to determine the methylation ratio of p21 and p27 promoters in T12752 CD133+ cells expressing Control shRNA, CD133 shRNA1, CD133 shRNA1 + shRNA-resistant (shR) wild-type CD133, or CD133 shRNA1 + shRNA-resistant CD133(1-862) mutant. Values are normalized to that of cells expressing control shRNA. Results are expressed as mean ± SD from three independent experiments; $***p < 0.001$, $**p < 0.01$, Student's t-test.

G. Western blot analysis of p21 and p27 protein level in CD133+ cells expressing Control shRNA, CD133 shRNA1, CD133 shRNA1+shRNA-resistant wild-type CD133, or CD133 shRNA1+shRNA-resistant CD133(1-862). GAPDH was used as a loading control. The relative densities of p21 or p27 to GAPDH were quantified using densitometry. Values are normalized to that of cells expressing control shRNA. Results are expressed as mean ± SD from three independent experiments; $***p <$

0.001, **$p$ < 0.01, Student's t-test.

H. Western blot analysis of DNMT1 protein level in CD133+ cells expressing control shRNA, DNMT1 shRNA1 or DNMT1 shRNA2. GAPDH was used as a loading control.

I. Bisulfite sequence assay was performed to determine the methylation level of p21 and p27 promoter in CD133+ cells expressing Control shRNA, CD133 shRNA1 and/or DNMT1 shRNA1 or DNMT1 shRNA2. Results are expressed as mean ± SD from three independent experiments; ***$p$ < 0.001, **$p$ < 0.01, Student's t-test.

Figure S4

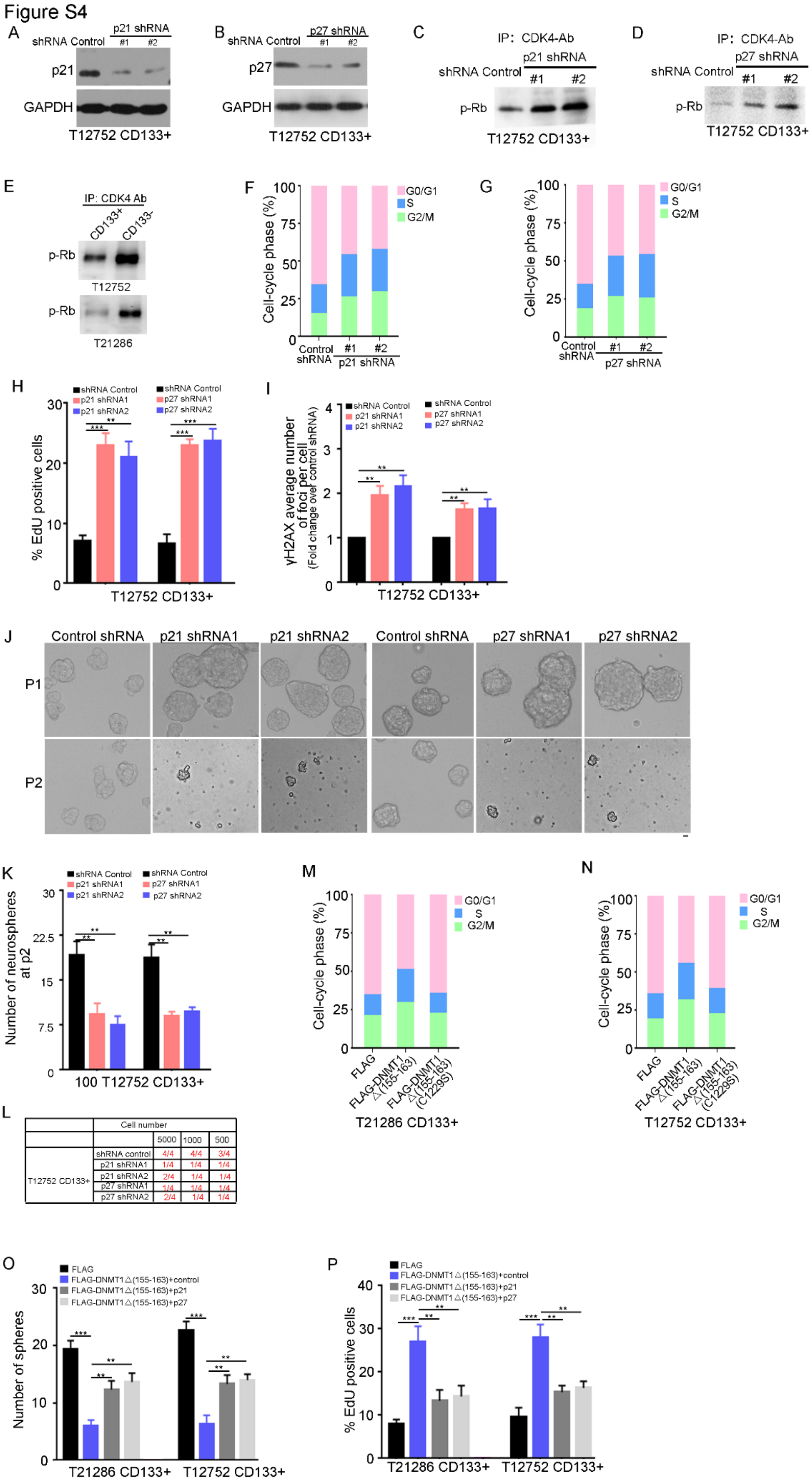


| | | Cell number | | |
|---|---|---|---|---|
| | | 5000 | 1000 | 500 |
| T12752 CD133+ | shRNA control | 4/4 | 4/4 | 3/4 |
| | p21 shRNA1 | 1/4 | 1/4 | 1/4 |
| | p21 shRNA2 | 2/4 | 1/4 | 1/4 |
| | p27 shRNA1 | 1/4 | 1/4 | 1/4 |
| | p27 shRNA2 | 2/4 | 1/4 | 1/4 |

**Figure S4. The nuclear localization of DNMT1 inhibits the self-renewal ability and tumorigenesis of glioma stem cells, related to Figure 4**

A-B. Western blot analysis of p21 (A) or p27 (B) protein level in CD133+ cells expressing the indicated shRNA. GAPDH was used as a loading control.

C-D. The lysates of CD133+ cells expressing control shRNA or p21 shRNA (C) or p27 shRNA (D) were prepared and immunoprecipitated with anti-CDK4 Ab. After washing, the immune complex-containing beads were analyzed for the associated kinase activity toward Rb.

E. The lysates of CD133+ cells and CD133- cells from T12752 (upper panel) and T21286 (lower panel) tissues were immunoprecipitated with antibodies to CDK4 as indicated. After washing, the immune complex-containing beads were analyzed for the associated kinase activity toward Rb.

F-G. Cell cycle distributions were determined by flow cytometry. Histogram shows the percentage of cells in G0/G1 phases (red), S phases (blue), and G2/M phases (green) of CD133+ cells expressing control shRNA and p21 shRNA (F) or p27 shRNA (G).

H. Analysis the percentage of EdU-positive cells in T12752 CD133+ cells expressing control shRNA, p21 shRNA, or p27 shRNA. Results are expressed as mean ± SD from six independent experiments; ***$p < 0.001$, **$p < 0.01$, Student's t-test.

I. Immunofluorescence analysis of γH2AX foci formation in CD133+ cells expressing Control shRNA or p21 shRNA or p27 shRNA. The number of γH2AX foci-positive cells was measured. Results are expressed as mean ± SD from three independent experiments; **$p < 0.01$, Student's t-test.

J-K. The number of spheres derived from 100 CD133+ cells expressing control shRNA or p21 shRNA or p27 shRNA at passages 1 and 2 were counted. J. Representative images were shown. K. Results are expressed as mean ± SD from three independent experiments; **$p < 0.01$, Student's t-test. Scale bar, 10 μM.

L. An intracranial limiting dilution tumor formation assay (employing 5,000, 1,000, and 500 cells per mouse) was performed using CD133+ cells infected with the indicated lentivirus. The table displays the number of mice developing tumors.

M-N. Cell cycle distributions were determined by flow cytometry. Histogram shows the percentage of cells in G0/G1 phases (red), S phases (blue), and G2/M phases (green) of T21286 (M) and T12752 (N) CD133+ cells expressing FLAG, FLAG-DNMT1(Del(155-163)) or its mutant.

O. The number of spheres derived from CD133+ cells expressing FLAG, FLAG-DNMT1(Del(155-163)), and p21 or p27. Results are expressed as mean ± SD from three independent experiments; **$p < 0.01$, ***$p < 0.001$, Student's t-test.

P. Immunofluorescence analysis of EdU-positive cekks in CD133+ cells expressing FLAG, FLAG-DNMT1(Del(155-163)), and p21 or p27. Results are expressed as mean ± SD from three independent experiments; ***$p < 0.001$, **$p < 0.01$, Student's t-test.

Figure S5

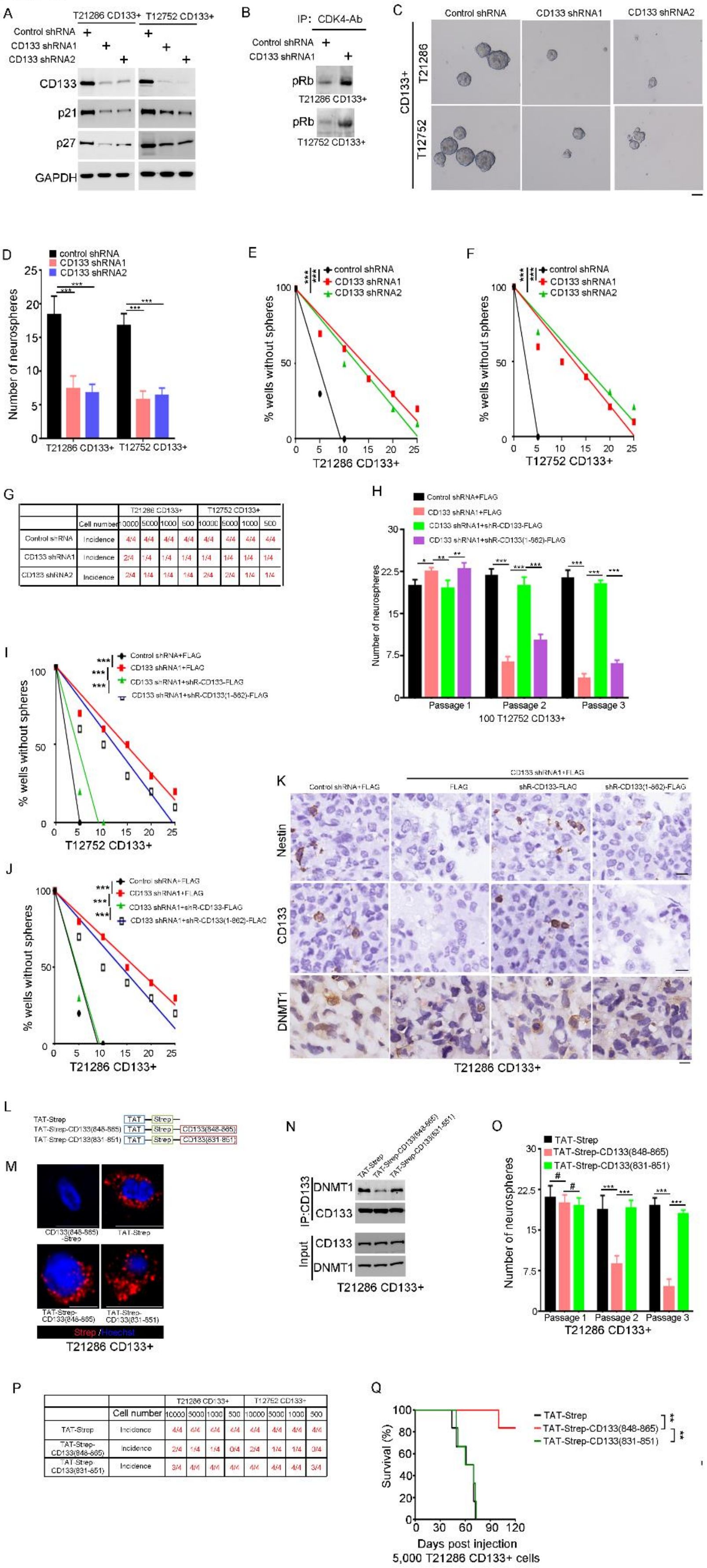


G

| | | T21286 CD133+ | | | | T12752 CD133+ | | | |
|---|---|---|---|---|---|---|---|---|---|
| | Cell number | 10000 | 5000 | 1000 | 500 | 10000 | 5000 | 1000 | 500 |
| Control shRNA | Incidence | 4/4 | 4/4 | 4/4 | 4/4 | 4/4 | 4/4 | 4/4 | 4/4 |
| CD133 shRNA1 | Incidence | 2/4 | 1/4 | 1/4 | 1/4 | 1/4 | 1/4 | 1/4 | 1/4 |
| CD133 shRNA2 | Incidence | 2/4 | 1/4 | 1/4 | 1/4 | 2/4 | 2/4 | 1/4 | 1/4 |

P

| | | T21286 CD133+ | | | | T12752 CD133+ | | | |
|---|---|---|---|---|---|---|---|---|---|
| | Cell number | 10000 | 5000 | 1000 | 500 | 10000 | 5000 | 1000 | 500 |
| TAT-Strep | Incidence | 4/4 | 4/4 | 4/4 | 4/4 | 4/4 | 4/4 | 4/4 | 4/4 |
| TAT-Strep-CD133(848-865) | Incidence | 2/4 | 1/4 | 1/4 | 0/4 | 2/4 | 1/4 | 1/4 | 0/4 |
| TAT-Strep-CD133(831-851) | Incidence | 3/4 | 4/4 | 4/4 | 4/4 | 4/4 | 4/4 | 4/4 | 3/4 |

**Figure S5. The CD133-DNMT1 interaction maintains the self-renewal capacity of glioma stem cell, related to Figure 5**

A. Western blot analysis of CD133, p21 and p27 expression in CD133+ cells expressing control shRNA and CD133 shRNA1-2. GAPDH expression served as a loading control.

B. The lysates of CD133+ cells expressing control shRNA or CD133 shRNA1 were prepared and immunoprecipitated with anti-CDK4 Ab. After washing, the immune complex-containing beads were analyzed for the associated kinase activity toward Rb.

C-D. A total of 100 T12752 CD133+ cells expressing Control shRNA，CD133 shRNA1, CD133 shRNA2 were cultured in plates. (C) Representative images of neurosphere at passage 2 are shown. Scale bar represents 50 µM. (D) Results are expressed as mean ± SD from three independent experiments; ***$p < 0.001$, Student's t-test.

E-F. Limiting dilution assay shows that CD133 knock down leads to reduced stem cell frequency in T21286 (E) or T12752 (F) CD133+ cells. n=10, ***$p < 0.001$ by ELDA analysis.

G. The tumor-initiating capacity of CD133+ cells expressing control shRNA, CD133 shRNA1 and CD133 shRNA2. An intracranial limiting dilution tumor formation assay (employing 10,000, 5,000, 1,000, and 500 cells per mouse) was performed using CD133+ cells infected with the indicated lentivirus. The table displays the number of mice developing tumors.

H. Single cell neurosphere formation assay of CD133+ cells expressing control shRNA，CD133 shRNA1, CD133 shRNA1+shRNA-resistant wild type CD133, or CD133 shRNA1+shRNA-resistant CD133(1-862) at passages 1-3. The number of neurosphere was shown. Results are expressed as mean ± SD from three independent experiments; ***$p < 0.001$, **$p < 0.01$, * $p < 0.05$, Student's t-test.

I-J. Limiting dilution assay shows CD133 knock down leads to reduced stem cell frequency in T12752 (I) or T21286 (J) CD133+ cells, which could be recued by wtCD133, not by CD133(1-862). n=10, ***$p < 0.001$ by ELDA analysis.

K. IHC staining for Nestin, CD133 and DNMT1 in xenograft formed by CD133+ cells expressing Control shRNA，CD133 shRNA1, CD133 shRNA1+shRNA-resistant wild type CD133, or CD133 shRNA1+shRNA-resistant CD133(1-862). Scale, 10 µM.

L. Graphical representation shows the structure of TAT-step-CD133(831-851) or

TAT-step-CD133(848-865). The cell-penetrating peptide TAT is in blue. The CD133 c-terminal is in Red. The linker between TAT and CD133 c-terminal is Strep (in green).

M. Immunofluorescence analysis of Strep-tagged protein in CD133+ glioma cell incubated for 1 hour with the indicated peptide (200 nM). Cells were washed, fixed and stained with anti-Strep (red) and Hoechst33258 (blue). Scale bars, 10 µM.

N. Co-IP assay was performed to determine the effect of peptides on the CD133-DNMT1 interaction. The lysates of CD133+ cells treated with the indicated peptide were subjected to IP using anti-CD133 Ab, followed by IB with anti-CD133 Ab or anti-DNMT1 Ab.

O. A total of 100 CD133+ cells isolated from T21286 sample were cultured in plates treated with the indicated peptides. The number of neurosphere at passages 1-3 was counted. Results are expressed as mean ± SD from three independent experiments; ***$p < 0.001$; #, ns. Student's t-test.

P. TAT-step-CD133(848-865) inhibited the tumor-initiating capacity of CD133+ cells. An intracranial limiting dilution tumor formation assay (employing 10,000, 5,000, 1,000, and 500 cells per mouse) was performed using CD133+ cells treated with the indicated peptides. The table displays the number of mice developing tumors.

Q. CD133+ cells from glioblastoma specimen T21286 treated with the indicated peptides were intracranially implanted into immunocompromised mice brain (5,000 cells per mouse). Mice were sacrificed when they were moribund or 120 days after implantation. Survival of mice (n = 6) was evaluated by Kaplan-Meier analysis (**$p < 0.01$, log rank test).

Figure S6

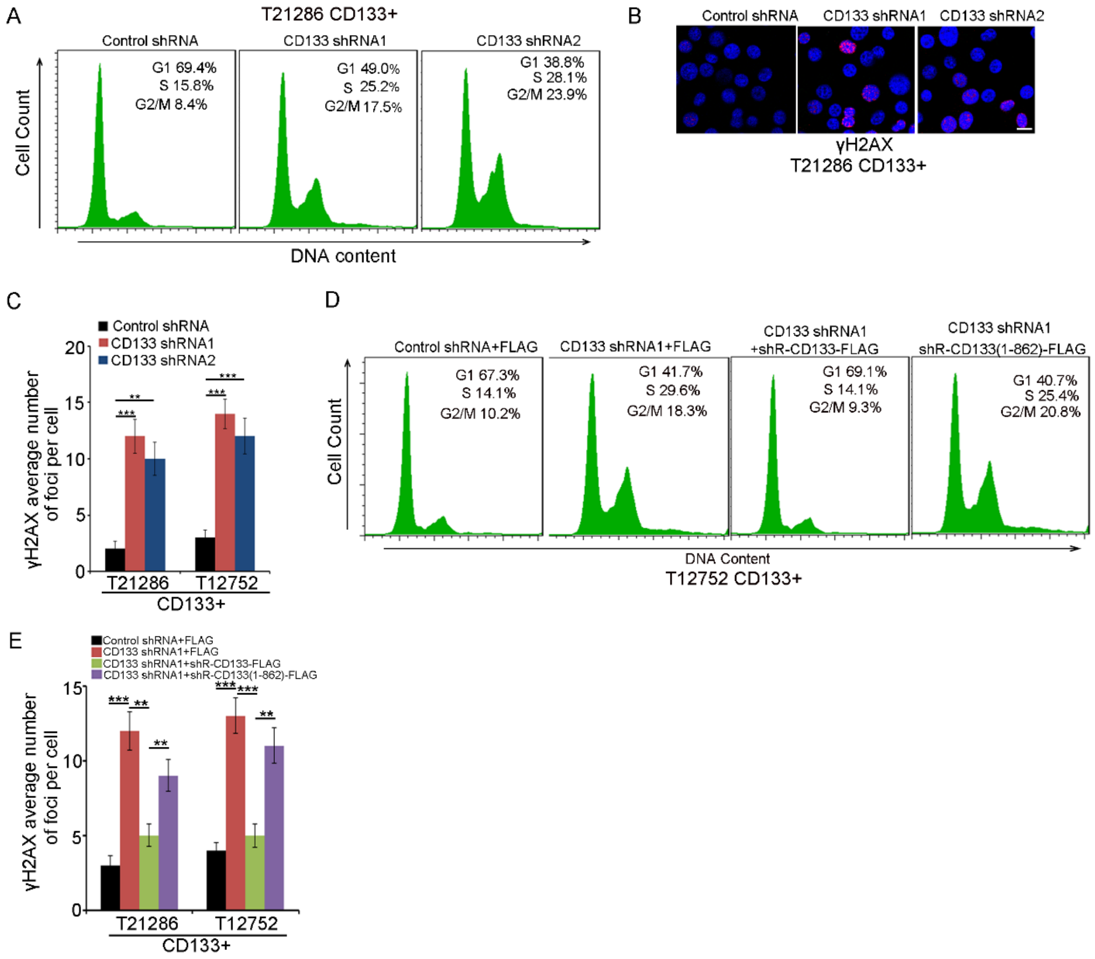


**Figure S6. The effect of the interaction between CD133 and DNMT1 on the DNA damage in glioma stem cell, related to Figure 6**

A. Cell cycle distributions of CD133+ cells expressing control shRNA or CD133 shRNA were determined by flow cytometry of PI-stained cells

B-C. CD133 knockdown increased the DNA damage in glioma stem cell. Immunofluorescence analysis of γH2AX foci formation in CD133+ cells expressing Control shRNA, CD133 shRNA1, or CD133 shRNA2. (B) Representative images of immunofluorescence are shown. (C) The number of γH2AX foci was measured. Results are expressed as mean ± SD from three independent experiments; ***$p$ < 0.001, **$p$ < 0.01. Student's t-test. Scale bar represents 10 μM.

D. Cell cycle distributions of CD133+ cells expressing Control shRNA，CD133 shRNA1, CD133 shRNA1+shRNA-resistant wild type CD133, or CD133 shRNA1+shRNA-resistant CD133(1-862) shRNA were determined by flow cytometry.

E. Immunofluorescence analysis the percentage of γH2AX foci formation in CD133+

cells expressing control shRNA，CD133 shRNA1, CD133 shRNA1+shRNA-resistant wild type CD133, or CD133 shRNA1+shRNA-resistant CD133(1-862). Results are expressed as mean ± SD from three independent experiments; ***$p < 0.001$, **$p < 0.01$, Student's t-test.

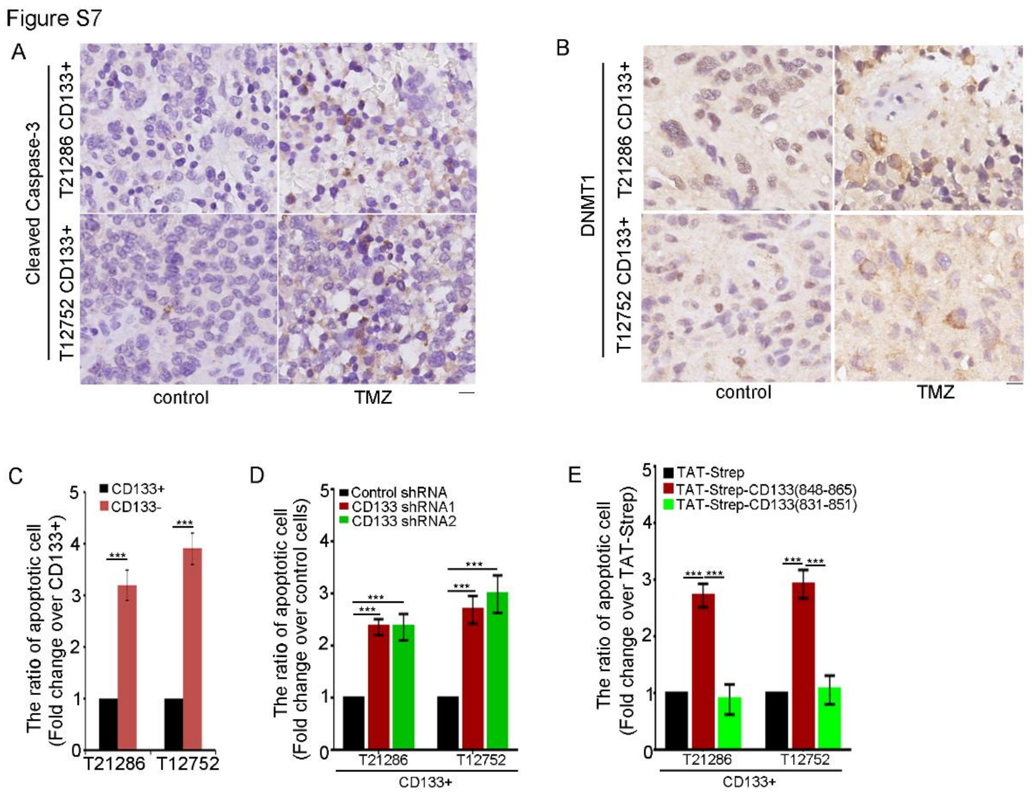


**Figure S7. The effect of the interaction between CD133 and DNMT1 on the apoptosis of glioma stem cell induced by TMZ, related to Figure 7**

A-B. IHC staining for cleaved-Caspase 3 (A) and DNMT1 (B) in xenograft formed by CD133+ cells treated with or without temozolomide. Scale, 10 μM.

C. CD133+ cells or CD133- cells were treated for 48 hours with temozolomide (200 μM). The ratio of apoptotic cells was measured by flow cytometry. Values are normalized to that of CD133+ cells. Results are expressed as mean ± SD from six independent experiments; ***$p < 0.001$.

D. CD133+ cells expressing Control shRNA，CD133 shRNA1, or CD133 shRNA2 were treated for 48 hours with temozolomide. The ratio of apoptotic cells was measured by flow cytometry. Values are normalized to that of cells expressing Control shRNA. Results are expressed as mean ± SD from six independent experiments; ***$p < 0.001$.

E. CD133+ cells from glioblastoma specimen treated with the indicated peptides were treated with temozolomide. The ratio of apoptotic cells was measured by FACS. Values are normalized to that of control cells. Results are expressed as mean ± SD from six independent experiments; ***$p < 0.001$.

Figure S8

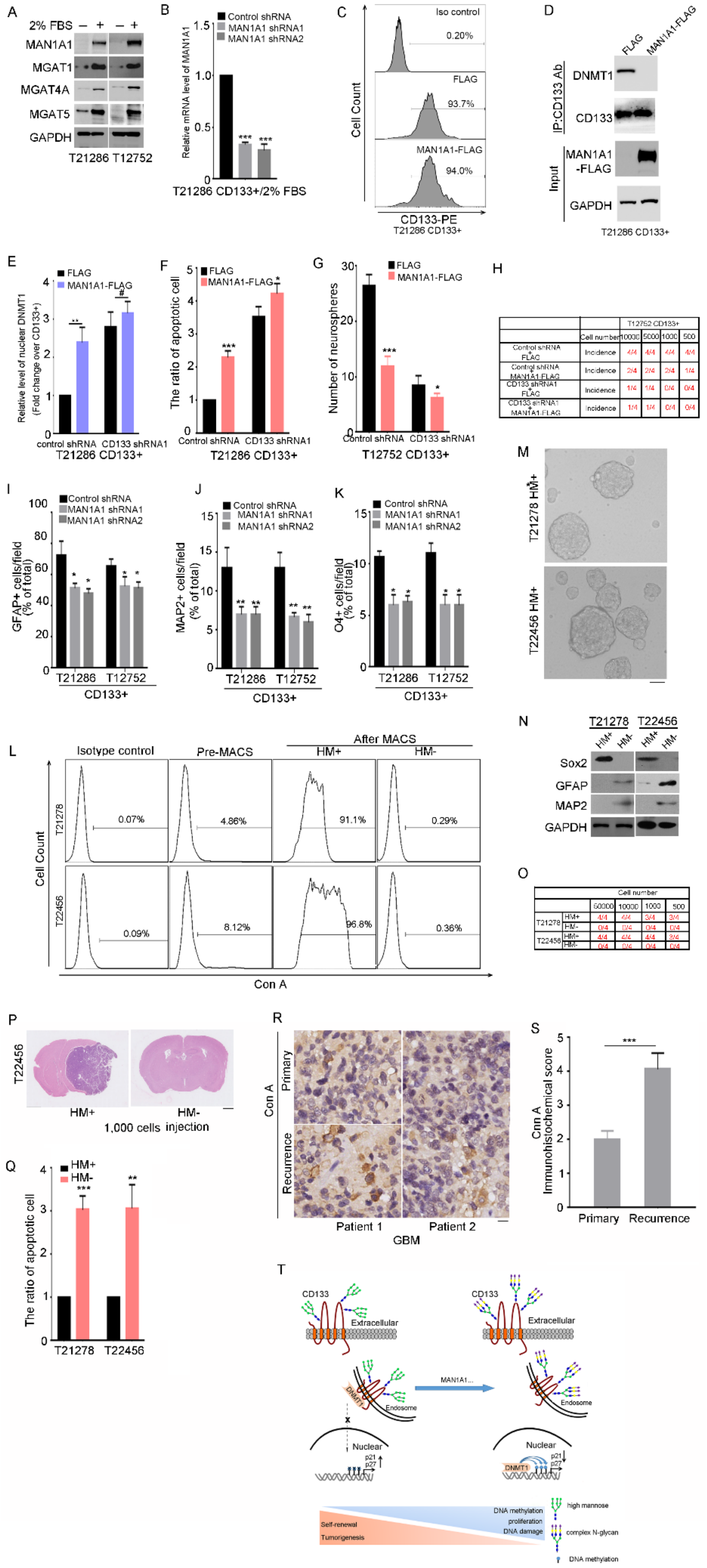


| | | T12752 CD133+ | | | |
|---|---|---|---|---|---|
| | Cell number | 10000 | 5000 | 1000 | 500 |
| Control shRNA + FLAG | Incidence | 4/4 | 4/4 | 4/4 | 4/4 |
| Control shRNA + MAN1A1-FLAG | Incidence | 2/4 | 2/4 | 2/4 | 1/4 |
| CD133 shRNA1 + FLAG | Incidence | 1/4 | 1/4 | 0/4 | 0/4 |
| CD133 shRNA1 + MAN1A1-FLAG | Incidence | 1/4 | 1/4 | 0/4 | 0/4 |

| | | Cell number | | | |
|---|---|---|---|---|---|
| | | 50000 | 10000 | 1000 | 500 |
| T21278 | HM+ | 4/4 | 4/4 | 3/4 | 3/4 |
| | HM- | 0/4 | 0/4 | 0/4 | 0/4 |
| T22456 | HM+ | 4/4 | 4/4 | 4/4 | 3/4 |
| | HM- | 0/4 | 0/4 | 0/4 | 0/4 |

**Figure S8. The high-mannose N-glycan of CD133 is necessary for its interaction with DNMT1, related to Figure 8**

A. Western blot analysis of the protein level of MAN1A1, MGAT1, MGAT4A, MGAT5 in CD133+ cells treated with or without 2% FBS for 7 days. GAPDH was used as a loading control.

B. QRT-PCR analysis of MAN1A1 expression in CD133+ cells treated with 2% FBS for 7 days expressing control shRNA or shMAN1A1-1 or shMAN1A1-2. Results are expressed as mean ± SD from three independent experiments; ***$p < 0.001$. Student's t-test.

C. FACS analysis of CD133 expression in CD133+ cells expressing control or MAN1A1-FLAG.

D. Co-IP assay was performed to evaluate the effect of MAN1A1 overexpression on the interaction between CD133 and DNMT1 in vivo. The lysates of CD133+ cells expression control or MAN1A1-FLAG were subjected to IP using anti-CD133 Ab (clone AC133), followed by IB with anti-CD133 or anti-DNMT1 Ab. Whole-cell lysates were analyzed by IB with anti-FLAG Ab as input.

E. Nuclear distribution of DNMT1 in CD133+ cells expressing control or MAN1A1-FLAG was determined by immunoblotting. The relative densities of DNMT1 to Histone H3 were quantified using densitometry. Values are normalized to that of control cells. Results are expressed as mean ± SD from three independent experiments; **$p < 0.01$, #, ns. Student's t-test.

F. CD133+ cells expressing control shRNA and/or CD133 shRNA1 and FLAG or MAN1A1-FLAG were treated for 48 hours with temozolomide. The ratio of apoptotic cells was measured by flow cytometry. Results are expressed as mean ± SD from three independent experiments; ***$p < 0.001$, *$p < 0.05$. Student's t-test.

G. A total of 100 CD133+ cells expressing control shRNA and/or CD133 shRNA1 and FLAG or MAN1A1-FLAG were cultured in plates. The number of neurosphere at passage 2 was counted. Results are expressed as mean ± SD from three independent experiments; ***$p < 0.001$, *$p < 0.05$. Student's t-test.

H. An in vivo limiting dilution tumor formation assay (employing 50,000, 10,000, 1,000 or 500 cells per mouse) was performed to compare the tumor-initiating capacity of CD133+ cells expressing control shRNA and/or CD133 shRNA1 and FLAG or MAN1A1-FLAG. Mice were sacrificed when they were moribund or 180 days after implantation. Tumor formation was determined by histology.

I-K. Multilineage differentiation capacity in CD133+ cells expressing control shRNA MAN1A1 shRNA1, or MAN1A2 shRNA2 was evaluated by staining for astrocytic (GFAP) (I), neuronal (MAP2) (J), and oligodendrocytic (O4) (K) markers. The positive cells were measured. Results are expressed as mean ± SD from three independent experiments; $^{**}p < 0.01$, $^{*}p < 0.05$. Student's t-test.

L. FACS analysis of the level of high mannose glycan on cell surface before and after sorting of HM+ and HM− fractions. HM+ cells were isolated from freshly dissociated GBM samples (T21278, T22456) by magnetic beads. The percentage of HM-positive cells was determined by FACS analysis. HM, high mannose N-glycan.

M. Representative image of neurospheres from HM+ cells isolated from glioblastoma specimens (T21278, T22456). Scale bar represents 10 µM.

N. Western blot analysis of stem cell-related genes and differentiation genes in HM+ cells and HM- cells.

O-P. An in vivo limiting dilution tumor formation assay (employing 50,000, 10,000, 1,000 or 500 cells per mouse) was performed to compare the tumor-initiating capacity of HM+ glioma cells with $HM^-$ glioma cells. Mice were sacrificed when they were moribund or 180 days after implantation. Tumor formation was determined by histology. O. The table displays number of mice developing tumors. P. H&E staining of mouse brain shows tumors formation by 5,000 HM+ but not by 5000 HM- cells. Scale bar represents 1 cm.

Q. HM+ cells and HM- cells were treated for 48 hours with temozolomide. The ratio of apoptotic cells was measured by flow cytometry. Results are expressed as mean ± SD from three independent experiments; $^{***}p < 0.001$. $^{**}p < 0.01$. Student's t-test.

R-S. IHC analysis of high mannose N-glycan in 16 paired primary and recurrent glioma sections. (R) Representative microphotographs of immunohistochemical staining of Con A in 16 paired primary and recurrent glioma sections. Scale bar represents 10 µM. (H) The scores for quantitative staining of high mannose (Con A positive) in the tissue sections were determined according to a total score (range, 0–8). $^{***}p < 0.001$, Values are mean ± SD (n = 16). Student's t-test.

T. Model of the CD133-DNMT1 interaction promoting the self-renewal and tumorigenesis of glioma stem cell. The lower expression of MAN1A1 results in the formation of high-mannose type N-glycan of CD133 in GSCs. The interaction between high-mannose CD133 and DNMT1 blocks the nuclear translocation of DNMT1. Activation of p21 and p27 expression by the CD133-DNMT1 interaction

maintains glioma stem cell quiescence, self-renewal, chemotherapy resistance and tumorigenesis.

**Table S1. Screening protein interacting with CD133 c-terminal (aa813-865) using the yeast two hybrid system, related to Figure 1**

C-terminal cytoplasmic domain of CD133 (residues 813-865) was used as the bait for yeast two-hybrid screen. 6 positive clones were obtained from at least $1 \times 10^6$ clones of a human fetal brain library. Among the 4 positive clones, 3 clones encoded partial sequences of DNMT1, and one clone encoded the sequence of COPS5, HSPA5 or ATP5B.

| Clone | Gene ID | Gene Name | Domain | Gene Function | Validation by IP in GSC |
|---|---|---|---|---|---|
| 1-3 | NM_003211.4 | DNMT1 | 112-235 | DNA Methyltransferase | Yes |
| 4 | NM_006837.2 | COPS5 | 92-200 | regulator of the ubiquitin | Yes |
| 5 | NM_005347.4 | HSPA5 | 72-150 | Heat shock protein | No |
| 6 | NM_001659.2 | ATP5B | 22-230 | a subunit of mitochondrial ATP synthase | No |

**Table. S2. Pathologic and Cytogenetic Characteristics of Brain Tumors, related to Figure 1**

Patient and pathological information associated with brain tumor samples is provided. The age and gender of patients as well as the tumor histopathology and TCGA subtype are included. PN, proneural; MES, Mesenchymal; NL, Neural. WT, wild type; LOSS, deletion; MUT, mutation.

| **Tumor designation** | **Pathologic Histology** | **Subtype** | **Primary/ recurrence** | **Tumor features** | |
|---|---|---|---|---|---|
| | | | | **PTEN** | **P53** |
| T21286 | Glioblastoma | NL | Primary | WT | WT |
| T12752 | Glioblastoma | PN | Primary | WT | WT |
| T08492 | Glioblastoma | MES | Primary | LOSS | WT |
| T21278 | Glioblastoma | PN | Primary | WT | WT |
| T22456 | Glioblastoma | PN | Primary | WT | WT |

**Table. S3.** Gene ontology results for 680 genes which methylation is upregulated in CD133+ cells expressing FLAG-DNMT1(Del(155-163)) were shown ($p < 0.05$), **related to Figure 3**

| **Term** | **Count** | **%** | **p Value** | **Fold Enrichment** | **Bonferroni** | **Benjamini** | **FDR** |
|---|---|---|---|---|---|---|---|
| GO:0007050~cell cycle arrest | 18 | 1.962922574 | 1.74E-04 | 2.869692 | 0.38273 | 0.338706 | 0.338706 |
| GO:0048009~insulin-like growth factor receptor signaling pathway | 6 | 0.654307525 | 2.44E-04 | 9.633964 | 0.492112 | 0.338706 | 0.338706 |
| GO:0098609~cell-cell adhesion | 24 | 2.617230098 | 0.002304295 | 1.990782 | 0.998326 | 1 | 1 |
| GO:0035264~multicellular organism growth | 11 | 1.199563795 | 0.002791974 | 3.090897 | 0.999568 | 1 | 1 |
| GO:0015031~protein transport | 31 | 3.380588877 | 0.003073773 | 1.764194 | 0.999803 | 1 | 1 |
| GO:0008360~regulation of cell shape | 15 | 1.635768811 | 0.003809763 | 2.408491 | 0.999975 | 1 | 1 |
| GO:0048010~vascular endothelial growth factor receptor signaling pathway | 10 | 1.090512541 | 0.004467167 | 3.122118 | 0.999996 | 1 | 1 |
| GO:0007399~nervous system development | 24 | 2.617230098 | 0.004716046 | 1.879798 | 0.999998 | 1 | 1 |
| GO:0006974~cellular response to DNA damage stimulus | 19 | 2.071973828 | 0.005391449 | 2.053393 | 1 | 1 | 1 |
| GO:0070141~response to UV-A | 3 | 0.327153762 | 0.005738711 | 22.47925 | 1 | 1 | 1 |
| GO:0007010~cytoskeleton organization | 15 | 1.635768811 | 0.012653977 | 2.09434 | 1 | 1 | 1 |
| GO:0060444~branching involved in mammary gland duct | 4 | 0.436205016 | 0.014221674 | 7.493083 | 1 | 1 | 1 |

| | | | | | | | |
|---|---|---|---|---|---|---|---|
| morphogenesis | | | | | | | |
| GO:0043065~positive regulation of apoptotic process | 23 | 2.508178844 | 0.015016876 | 1.723409 | 1 | 1 | 1 |
| GO:0030900~forebrain development | 7 | 0.763358779 | 0.015530697 | 3.420755 | 1 | 1 | 1 |
| GO:0046835~carbohydrate phosphorylation | 5 | 0.54525627 | 0.01744042 | 4.886794 | 1 | 1 | 1 |
| GO:0043547~positive regulation of GTPase activity | 37 | 4.034896401 | 0.019139088 | 1.472092 | 1 | 1 | 1 |
| GO:0006977~DNA damage response, signal transduction by p53 class mediator resulting in cell cycle arrest | 8 | 0.872410033 | 0.019609622 | 2.900548 | 1 | 1 | 1 |
| GO:0034968~histone lysine methylation | 4 | 0.436205016 | 0.022027567 | 6.422643 | 1 | 1 | 1 |
| GO:0070372~regulation of ERK1 and ERK2 cascade | 5 | 0.54525627 | 0.023236985 | 4.49585 | 1 | 1 | 1 |
| GO:0006066~alcohol metabolic process | 3 | 0.327153762 | 0.026243089 | 11.23963 | 1 | 1 | 1 |
| GO:0007213~G-protein coupled acetylcholine receptor signaling pathway | 4 | 0.436205016 | 0.026643975 | 5.994467 | 1 | 1 | 1 |
| GO:0042692~muscle cell differentiation | 4 | 0.436205016 | 0.026643975 | 5.994467 | 1 | 1 | 1 |
| GO:0034097~response to cytokine | 7 | 0.763358779 | 0.02703403 | 3.026053 | 1 | 1 | 1 |
| GO:0007223~Wnt signaling pathway, calcium modulating | 6 | 0.654307525 | 0.028184435 | 3.458346 | 1 | 1 | 1 |

| | | | | | | | |
|---|---|---|---|---|---|---|---|
| pathway | | | | | | | |
| GO:0007067~mitotic nuclear division | 19 | 2.071973828 | 0.028448069 | 1.722201 | 1 | 1 | 1 |
| GO:0043010~camera-type eye development | 6 | 0.654307525 | 0.031073891 | 3.371888 | 1 | 1 | 1 |
| GO:0061036~positive regulation of cartilage development | 4 | 0.436205016 | 0.0317347 | 5.619813 | 1 | 1 | 1 |
| GO:0050690~regulation of defense response to virus by virus | 5 | 0.54525627 | 0.03389234 | 4.014152 | 1 | 1 | 1 |
| GO:0006468~protein phosphorylation | 30 | 3.271537623 | 0.033962245 | 1.478898 | 1 | 1 | 1 |
| GO:0007605~sensory perception of sound | 12 | 1.308615049 | 0.034810441 | 2.028203 | 1 | 1 | 1 |
| GO:0007265~Ras protein signal transduction | 8 | 0.872410033 | 0.03539062 | 2.569057 | 1 | 1 | 1 |
| GO:0008064~regulation of actin polymerization or depolymerization | 3 | 0.327153762 | 0.035671441 | 9.633964 | 1 | 1 | 1 |
| GO:0008283~cell proliferation | 25 | 2.726281352 | 0.03736818 | 1.535468 | 1 | 1 | 1 |
| GO:0007595~lactation | 6 | 0.654307525 | 0.037392195 | 3.211321 | 1 | 1 | 1 |
| GO:0000082~G1/S transition of mitotic cell cycle | 10 | 1.090512541 | 0.037559738 | 2.203848 | 1 | 1 | 1 |
| GO:0097193~intrinsic apoptotic signaling pathway | 5 | 0.54525627 | 0.042335708 | 3.746542 | 1 | 1 | 1 |
| GO:0051017~actin filament bundle assembly | 5 | 0.54525627 | 0.042335708 | 3.746542 | 1 | 1 | 1 |
| GO:1903076~regulation of protein localization to | 3 | 0.327153762 | 0.04618362 | 8.429719 | 1 | 1 | 1 |

| | | | | | | | |
|---|---|---|---|---|---|---|---|
| plasma membrane | | | | | | | |
| GO:0042698~ovulation cycle | 3 | 0.327153762 | 0.04618362 | 8.429719 | 1 | 1 | 1 |
| GO:0014909~smooth muscle cell migration | 3 | 0.327153762 | 0.04618362 | 8.429719 | 1 | 1 | 1 |

**Table. S4. The methylation of genes regulating cell cycle progression are upregulated CD133+ cells expressing DNMT1 (Del(155-163)) ($p < 0.001$), related to Figure 3**

| ACCESSION | NAME | Beta.Difference | P.Value | UCSC_REFGENE_GROUP |
|---|---|---|---|---|
| NM_000389 | CDKN1A | 0.186168073 | 0.00012345 | TSS200 |
| NM_004064 | CDKN1B | 0.136168073 | 0.0004562 | TSS200 |
| NM_024408 | NOTCH2 | 0.151558806 | 0.000181151 | Body |
| NM_001164766 | ZFHX3 | 0.186433004 | 0.000245587 | 5'UTR |
| NM_001130849 | CAB39 | 0.155035679 | 0.000467775 | Body |
| NM_133646 | ZAK | 0.153985296 | 0.000267795 | Body |
| NM_014909 | VASH1 | 0.161342123 | 0.000306225 | Body |
| NM_033244 | PML | 0.184233699 | 0.000542256 | Body |
| NM_003589 | CUL4A | 0.170558246 | 3.83E-05 | Body |
| NM_001163034 | RPTOR | 0.189640703 | 0.000697841 | Body |
| NM_001163034 | RPTOR | 0.162701754 | 0.00016472 | Body |
| NM_001163034 | RPTOR | 0.157222573 | 0.000103785 | Body |
| NM_005146 | SART1 | 0.150040925 | 0.000133851 | Body |
| NM_003884 | KAT2B | 0.195693696 | 0.000381004 | Body |
| NM_000038 | APC | 0.174923105 | 2.43E-05 | 5'UTR |
| NM_005614 | RHEB | 0.167430367 | 8.39E-05 | Body |
| NM_002198 | IRF1 | 0.159072393 | 0.000179095 | Body |
| NM_001143830 | GAS2 | 0.153009025 | 3.82E-05 | Body |
| NM_001244262.1 | HBP1 | 0.155349678 | 4.68E-06 | Body |
| NM_000051 | ATM | 0.151516434 | 8.02E-05 | Body |

KEY RESOURCES TABLE

| REAGENT or RESOURCE | SOURCE | IDENTIFIER |
|---|---|---|
| **1. Antibody** | | |
| CD133(W6B3C1) pure | Miltenyi Biotec | Cat # 130-092-395 |
| CD133(AC133) pure | Miltenyi Biotec | Cat # 130-090-422 |
| CD133/1(AC133)-PE | Miltenyi Biotec | Cat # 130-080-801 |
| GFAP | Chemicon | Cat # AB5804 |
| MAP2 | Sigma | Cat # M4403 |
| O4 | Sigma | Cat # O7139 |
| Nestin | Chemicon | Cat # MAB5326 |
| Sox2 | CST | Cat #3579s |
| DNMT1 | Abcam | Cat #Ab13537 |
| GAPDH | Cell Signaling | Cat #5174 |
| β-actin | Sigma | Cat #A5441 |
| EEA1 | CST | Cat #3288T |
| GM130 | CST | Cat #12480 |
| Biotinylated Con A lectin | Sigma | Cat #C2272 |
| Biotinylated DSL lectin | VECTOR | Cat #B-1185 |
| Biotinylated PHA-L | VECTOR | Cat #B-1115 |
| MGAT1 | Abcam | Cat #Ab180578 |
| MGAT5 | R&D | Cat #MAB5469 |
| P21 | CST | Cat #2947 |
| P27 | CST | Cat #3686 |
| goat anti-rabbit IgG-HRP Secondary Antibody | Santa CruZ | Cat #Sc-2004 |
| goat anti-mouse IgG-HRP Secondary Antibody | Santa CruZ | Cat #Sc-2005 |
| Streptavidin-HRP | SoutherBiotech | Cat #7100-05 |
| Donkey anti-Goat IgG (H+L) Cross-Adsorbed, Alexa Fluor® 647 | Invitrogen | Cat #A21447 |
| Donkey anti-Rabbit IgG (H+L) Highly Cross-Adsorbed, Alexa Fluor® 647 | Invitrogen | Cat #A31573 |
| Donkey anti-Rabbit IgG (H+L) Highly Cross-Adsorbed, Alexa Fluor® 488 | Invitrogen | Cat #A21206 |
| Donkey anti-Mouse IgG (H+L) | Invitrogen | Cat #A21202 |

| | | |
|---|---|---|
| Highly Cross-Adsorbed, Alexa Fluor® 488 | | |
| Donkey anti-Goat IgG (H+L) Cross-Adsorbed, Alexa Fluor® 488 | Invitrogen | Cat #A11055 |
| Donkey anti-Mouse IgG (H+L) Highly Cross-Adsorbed, Alexa Fluor® 594 | Invitrogen | Cat #A21203 |
| Donkey anti-Rabbit IgG (H+L) Highly Cross-Adsorbed, Alexa Fluor® 594 | Invitrogen | Cat #A21207 |
| **2. Chemicals, Peptides, and Recombinant Proteins** | | |
| B27 | Gbico | Cat #12587-010 |
| DMEM/F12 | Gbico | Cat #11320082 |
| Fetal Bovine Serum | Gbico | Cat #10099-141 |
| DMEM | Gbico | Cat #11995073 |
| DPBS, powder, no calcium, no magnesium | Gbico | Cat #21600-069 |
| Matrigel | BD Biosciences | Cat #356234 |
| EGF | Merck Millipore | Cat #GF144 |
| FGF | Merck Millipore | Cat #GF003 |
| MS Columns | Miltenyi Biotec | Cat #130-042-201 |
| LS Columns | Miltenyi Biotec | Cat #130-042-401 |
| Protein marker | Thermo | Cat #26617 |
| Cooktail | Merck | Cat #539134 |
| Power SYBR™ Green PCR Master Mix | Applied Biosystems™ | Cat #4367659 |
| Lipo2000 | Invitrogen™ | Cat #11668-019 |
| Fluorescence Mounting Medium | DAKO | Cat #S3023 |
| **3. Experimental Models: Organisms/Strains** | | |
| NOD-SCID | Beijing Vital River Laboratory Animal Technology Co., Ltd. | |
| **4. Recombinant DNA** | | |
| LentiCRISPR v2 | Addgene | Plasmid # 52961 |
| **5. Critical Commercial Assays** | | |
| MethylFlash Global DNA Methylation (5-mC) ELISA Easy Kit | Epigentek | Cat #P-1030 |
| SimpleChIP® Plus Enzymatic Chromatin IP Kit | Cell signaling | Cat #9004 |
| CD133 Micro bead kit | Miltenyi Biotec | Cat #130-050-801 |
